# 3‐HKA Promotes Vascular Remodeling After Stroke by Modulating the Activation of A1/A2 Reactive Astrocytes

**DOI:** 10.1002/advs.202412667

**Published:** 2025-01-24

**Authors:** Jun‐Min Chen, Guang Shi, Lu‐Lu Yu, Wei Shan, Jing‐Yu Sun, An‐Chen Guo, Jian‐Ping Wu, Tie‐Shan Tang, Xiang‐Jian Zhang, Qun Wang

**Affiliations:** ^1^ Department of Neurology Beijing Tiantan Hospital Capital Medical University Beijing 100070 China; ^2^ Department of Neurology Second Hospital of Hebei Medical University Shijiazhuang 050000 China; ^3^ China National Clinical Research Center for Neurological Diseases Beijing 100070 China; ^4^ Hebei Key Laboratory of Vascular Homeostasis and Hebei Collaborative Innovation Center for Cardio‐cerebrovascular Disease Shijiazhuang 050000 China; ^5^ Beijing Institute of Brain Disorders Collaborative Innovation Center for Brain Disorders Capital Medical University Beijing 100069 China; ^6^ Key Laboratory of Organ Regeneration and Reconstruction State Key Laboratory of Membrane Biology Institute of Zoology Chinese Academy of Sciences Beijing 100101 China; ^7^ Beijing Institute for Stem Cell and Regenerative Medicine Beijing 100101 China; ^8^ University of Chinese Academy of Sciences Beijing 101408 China; ^9^ Beijing Key Laboratory of Drug and Device Research and Development for Cerebrovascular Diseases Beijing 100070 China; ^10^ Advanced Innovation Center for Human Brain Protection Capital Medical University Beijing 100070 China

**Keywords:** 3‐hydroxy‐kynurenamine, AIM2 inflammasomes, ischemic stroke, reactive astrocytes, vascular remodeling

## Abstract

Ischemic stroke is the most common cerebrovascular disease and the leading cause of permanent disability worldwide. Recent studies have shown that stroke development and prognosis are closely related to abnormal tryptophan metabolism. Here, significant downregulation of 3‐hydroxy‐kynurenamine (3‐HKA) in stroke patients and animal models is identified. Supplementation with 3‐HKA improved long‐term neurological recovery, reduced infarct volume, and increased ipsilateral cerebral blood flow after distal middle cerebral artery occlusion (MCAO). 3‐HKA promoted angiogenesis, functional blood vessel formation, and blood‐brain barrier (BBB) repair. Moreover, 3‐HKA inhibited A1‐like (neurotoxic) astrocyte activation but promoted A2‐like (neuroprotective) astrocyte polarization. Proteomic analysis revealed that 3‐HKA inhibited AIM2 inflammasome activation after stroke, and co‐labeling studies indicated that AIM2 expression typically increased in astrocytes at 7 and 14 days after stroke. Consistently, in co‐cultures of primary mouse brain microvascular endothelial cells and astrocytes, 3‐HKA promoted angiogenesis after oxygen‐glucose deprivation (OGD). AIM2 overexpression in astrocytes abrogated 3‐HKA‐driven vascular remodeling in vitro and in vivo, suggesting that 3‐HKA may regulate astrocyte‐mediated vascular remodeling by impeding AIM2 inflammasome activation. In conclusion, 3‐HKA may promote post‐stroke vascular remodeling by regulating A1/A2 astrocyte activation, thereby improving long‐term neurological recovery, suggesting that supplementation with 3‐HKA may be an efficient therapy for stroke.

## Graphical abstract

This study identifies 3‐hydroxy‐kynurenamine (3‐HKA) as a significantly downregulated tryptophan metabolite after ischemic stroke. Supplementation with 3‐HKA enhances long‐term neurological recovery by promoting vascular remodeling, through which the regulation of astrocyte A1/A2 transition is mediated by inhibiting AIM2 inflammasome activation. These findings reveal a novel mechanism how 3‐HKA supports recovery after stroke.

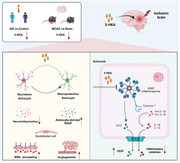

## 1 Introduction

Ischemic stroke is one of the most common causes of mortality and permanent disability worldwide, imposing a substantial burden on society and families. While the introduction of tissue plasminogen activator (tPA)‐mediated thrombolysis and endovascular thrombectomy has greatly increased treatment options for ischemic attacks during the acute time window, there are still limited therapeutic approaches available for improving long‐term post‐stroke recovery.^[^ ^1^, ^2^, ^3^, ^4^, ^5^ ^]^ Angiogenesis and restoration of the blood‐brain barrier (BBB) in the ischemic penumbra play important roles in stroke recovery. The kinetics of vascular remodeling significantly impact the recovery trajectory; for example, spontaneous recovery typically occurs in the initial weeks or months following a stroke. Recent evidence suggests that neovascularization after stroke is crucial for endogenous repair processes and that newly formed blood vessels can increase tissue perfusion in the peri‐infarct area, while also contributing to neural repair.^[^ ^1^, ^6^, ^7^, ^8^, ^9^ ^]^ Furthermore, increased microvascular density in the penumbra is associated with improved long‐term prognosis after stroke.^[^ ^3^ ^]^ Therefore, strengthening vascular remodeling after stroke is crucial for minimizing brain damage and improving patient prognosis.

Tryptophan and its metabolites, including serotonin, kynurenine, and indole, are crucial for the maintenance of physiological homeostasis.^[^ ^10^, ^11^, ^12^, ^13^ ^]^ Thus, tryptophan metabolites are emerging as promising therapeutic targets for conditions such as depression, Alzheimer's disease (AD), heart failure, colitis, and liver cancer.^[^ ^10^, ^14^, ^15^, ^16^, ^17^ ^]^ Recently, the occurrence and prognosis of ischemic stroke have been shown to be closely associated with abnormalities in tryptophan metabolism.^[^ ^18^, ^19^ ^]^ In this study, we found that in patients with acute ischemic stroke (AIS) and in experimental animal models, tryptophan metabolism was disrupted, accompanied by a considerable decrease in the levels of 3‐hydroxy‐kynurenamine (3‐HKA). 3‐HKA has been demonstrated to have a therapeutic effect in mouse models of psoriasis and nephrotoxic nephritis by inhibiting STAT1 and NF‐kB activation and reducing inflammatory responses.^[^ ^20^ ^]^ However, whether 3‐HKA could also aid in functional restoration after stroke is unknown.

In this study, we aimed to identify tryptophan metabolites with protective effects against ischemia in stroke patients and animal models and elucidate their mechanisms of action. We identified 3‐HKA to be significantly downregulated after stroke. Supplementation with 3‐HKA improved long‐term neurological recovery in ischemic stroke mice, reduced cerebral infarct volume, and increased ipsilateral cerebral blood flow (CBF). 3‐HKA supplementation promoted vascular remodeling in the peri‐infarct area. 3‐HKA also triggered a bolstering of A2‐like astrocytes while suppressing A1‐like astrocytes after stroke. Mechanistically, 3‐HKA facilitated endothelial cell proliferation by inhibiting the activation of AIM2 inflammasomes in astrocytes, modulating the activation of A1/A2 reactive astrocytes, reducing inflammatory cytokine release, and increasing astrocyte‐derived VEGF. Our findings indicated that 3‐HKA enhances vascular remodeling via regulation of the astrocyte A1/A2 transition by impeding AIM2 inflammasome activation, suggesting a previously overlooked mechanism by which 3‐HKA promotes recovery after stroke.

## 2 Results

### 2.1 Systemic 3‐HKA Levels were Reduced in Patients with AIS and in Experimental Animals

**Figure 1:**
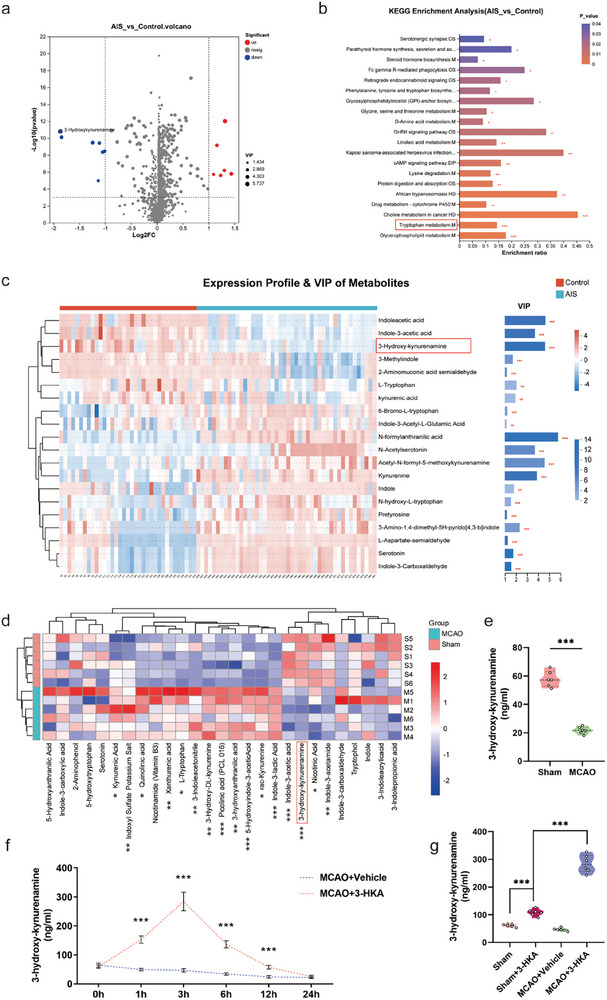
Identification of differentially abundant plasma metabolites associated with stroke. a) Volcano plot of metabolites with differential abundance in patients with AIS and healthy controls. b) KEGG pathway enrichment diagram of metabolites, with the abscissa indicating the enrichment ratio. *P < 0.05, **P < 0.01, ***P < 0.001, AIS group (n = 46) versus Control group (n = 35). c) Expression profile and variable importance in projection (VIP) metabolites between the AIS and control groups. *P < 0.05, **P < 0.01, ***P < 0.001, AIS group (n = 46) versus Control group (n = 35). d) Heatmap showing the hierarchical clustering and relative abundance of significantly differentiated tryptophan metabolites in the brain tissue of the mice in the MCAO and sham groups. *P < 0.05, **P < 0.01, ***P < 0.001, MCAO group versus Sham group, n = 6. e) Violin plot showing the relative differences in the levels of 3‐HKA in the brain tissues of the mice. ***P < 0.001, MCAO group versus Sham group, n = 6. f) Quantification of 3‐HKA levels in mouse brain tissue at individual time points after treatment. ***P < 0.001, MCAO+3‐HKA group versus MCAO+Vehicle group. Student's t test at individual time points. n = 6. g) Quantification of 3‐HKA levels in mouse brain tissue 3 h after treatment. ***P < 0.001, Sham+3‐HKA group versus Sham group; ***P < 0.001, MCAO+3‐HKA group versus Sham+3‐HKA group; one‐way ANOVA followed by Dunnett's multiple comparison tests. n = 6.

To monitor metabolic alterations after stroke, untargeted metabolomic analysis was performed on plasma samples from AIS patients and healthy controls. Using partial least squares discriminant analysis (PLS‐DA), we identified 602 metabolites with differential abundance between the AIS and healthy control groups, with 485 upregulated and 117 downregulated in the stroke group (**Figure** **1**a; Figure S1a, Supporting Information). Notably, the levels of tryptophan metabolites, including 3‐HKA, indoleacetic acid, kynurenic acid and L‐tryptophan, were severely disrupted after stroke (Figure 1b,c). Using orthogonal approaches, including calculation of fold changes (FCs), P values, variable influence in projection (VIP) values, Kyoto Encyclopedia of Genes and Genomes (KEGG) pathway analysis scores and biological relevance, we identified 3‐HKA as one of the top differentially abundant metabolites in the stroke group compared with the control group (FC = 0.274, *P* < 0.0001; Student's t test, VIP = 4.5765) (Figure 1c).

To further explore the alterations in tryptophan metabolism after stroke, we then performed targeted metabolomic analysis in a mouse model of cerebral ischemic injury. We found that 12 tryptophan metabolites, including indole‐3‐lactic acid, kynurenine, picolinic acid, and 3‐hydroxyanthranilic acid, were upregulated, whereas 4 metabolites, including 3‐HKA, nicotinic acid, and indole‐3‐acetic acid, were downregulated in mice after distal middle cerebral artery occlusion (MCAO) (Figure 1d,e; Figure S1b,c, Supporting Information). Together, these observations confirmed the downregulation of 3‐HKA in both AIS patients and experimental stroke model animals, suggesting that 3‐HKA may play crucial roles in stroke. To address the pharmacokinetics of 3‐HKA, we established the MCAO model and administered 3‐HKA (40 mg kg^−1^) immediately post‐stroke. We then measured the levels of 3‐HKA in the ischemic peri‐infarct brain tissue at various time points: pre‐treatment (0 h) and post‐treatment (1, 3, 6, 12, and 24 h). Our results indicated that in the MCAO+Vehicle group, 3‐HKA levels began to decline at 1 h post‐stroke and continued to decrease over the 24 h period (Figure 1f). Notably, in the MCAO+3‐HKA group, 3‐HKA levels in the brain tissue increased at 1 h post‐treatment, peaked at 3 h, and significantly decreased by 6 h (Figure 1f). Additionally, we assessed 3‐HKA levels in the brain tissue of healthy mice, revealing accumulation at 3 h post‐treatment. However, the concentration of 3‐HKA in the ischemic brain tissue of stroke mice at 3 h post‐treatment was significantly higher than that observed in healthy controls (Figure 1g). These results indicated that the administration of 3‐HKA preferentially accumulated in ischemic brain tissue, exerting potential biological effects.

### 2.2 3‐HKA Improved Long‐Term Neurological Recovery and Increased CBF after Stroke

**Figure 2:**
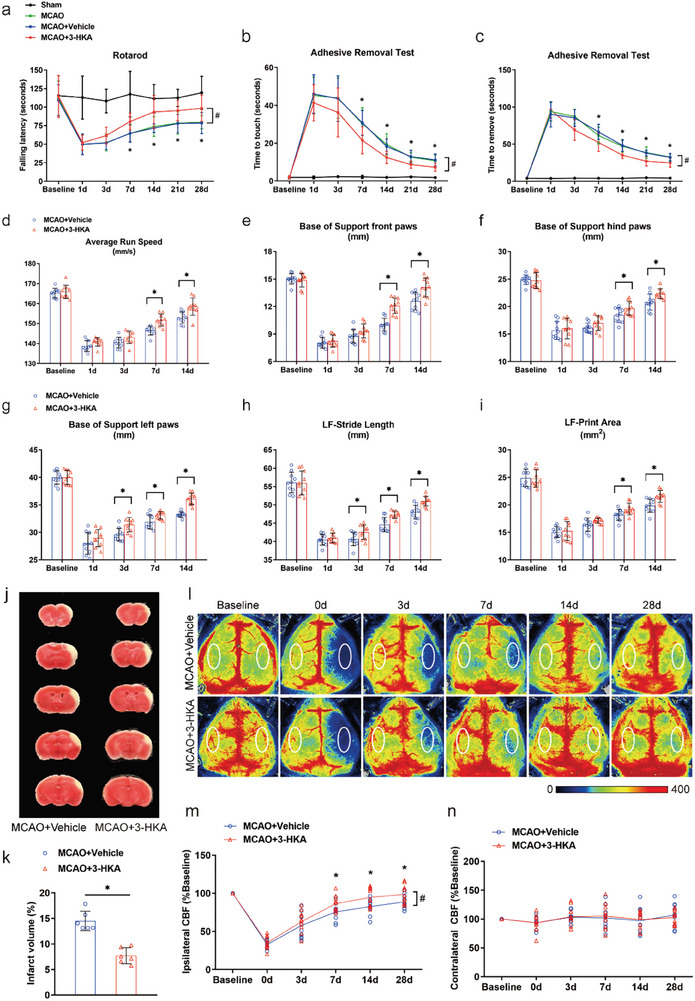
3‐HKA improved long‐term neurological recovery and enhanced cerebral blood flow. a) Rotarod test. *P* = 0.026171 at 7 d, *P* = 0.005 at 14 d, *P* = 0.004164 at 21 d, and *P* = 0.047938 at 28 d (MCAO+3‐HKA versus MCAO+Vehicle); one‐way ANOVA followed by LSD multiple comparison tests (21 d) or Dunnett's multiple comparison tests (7 d and 28 d). The Kruskal‐Wallis test followed by Dunn's post hoc analysis was performed at the 14 d time points. ^#^ *P* < 0.001 (MCAO+3‐HKA versus MCAO+Vehicle) by two‐way ANOVA followed by Bonferroni's multiple comparison tests (bracket). n = 10 for the Sham group; n = 11 for the MCAO, MCAO+Vehicle, and MCAO+3‐HKA groups. b) Time to touch the adhesive tape. *P* = 0.021 at 7 d, *P* = 0.006543 at 14 d, *P* = 0.014227 at 21 d, and *P* = 0.024 at 28 d (MCAO+3‐HKA versus MCAO+Vehicle); one‐way ANOVA followed by Dunnett's multiple comparison tests (14 d and 21 d). The Kruskal‐Wallis test followed by Dunn's post hoc analysis was performed at the 7 d and 28 d time points. ^#^ *P* < 0.001 (MCAO+3‐HKA versus MCAO+Vehicle) by two‐way ANOVA followed by Bonferroni's multiple comparison tests (bracket). n = 10 for the Sham group; n = 11 for the MCAO, MCAO+Vehicle, and MCAO+3‐HKA groups. c) Time to remove the adhesive tape. *P* = 0.040597 at 7 d, *P* =  0.001025 at 14 d, *P* = 0.0009 at 21 d, and *P* = 0.033521 at 28 d (MCAO+3‐HKA versus MCAO+Vehicle); one‐way ANOVA followed by Dunnett's multiple comparison tests. ^#^ *P* < 0.001 (MCAO+3‐HKA versus MCAO+Vehicle) by two‐way ANOVA followed by Bonferroni's multiple comparison tests (bracket). d) Average run speed measured in the gait test. *P* = 0.000258 at 7 d and *P* = 0.003383 at 14 d, MCAO+3‐HKA versus MCAO+Vehicle; Student's t test at individual time points. e) Base of support (front paws) measured in the gait test. *P* = 0.000010 at 7 d and *P* = 0.003557 at 14 d, MCAO+3‐HKA versus MCAO+Vehicle; Student's t test at individual time points. n = 10. f) Base of support (hind paws) measured in the gait test. *P* =  0.041958 at 7 d and *P* = 0.010978 at 14 d, MCAO+3‐HKA versus MCAO+Vehicle; Student's t test at individual time points. n = 10. g) Base of support (left paws) measured in the gait test. *P* = 0.00467 at 3 d, *P* =  0.020432 at 7 d, and *P* = 0.000003 at 14 d, MCAO+3‐HKA versus MCAO+Vehicle; Student's t test at individual time points. n = 10. h) Stride length for the left front paw. *P* = 0.042364 at 3 d, *P* = 0.001686 at 7 d, and *P* = 0.000643 at 14 d, MCAO+3‐HKA versus MCAO+Vehicle; Student's t test at individual time points. n = 10. i) Pawprint area for the left front paw. *P* =  0.036963 at 7 d and *P* = 0.003807 at 14 d, MCAO+3‐HKA versus MCAO+Vehicle; Student's t test at individual time points. n = 10. j) Representative TTC stained sections at day 7 after stroke. k) Effect of 3‐HKA on infarct volume 7 d after stroke. *P* = 0.000052, MCAO+3‐HKA versus MCAO+Vehicle; Student's t test. n = 6. l) Representative laser speckle images of CBF. Two identical elliptical ROIs were selected as indicated on the ipsilateral and contralateral hemispheres. m) Quantification of the ipsilateral CBF. *P =  0.029147 at 7 d, *P = 0.023798 at 14 d, and *P = 0.045707 at 28 d, MCAO+3‐HKA versus MCAO+Vehicle, Student's t test at individual time points. ^#^ *P* = 0.000375, MCAO+3‐HKA versus MCAO+Vehicle, by two‐way ANOVA followed by Bonferroni's multiple comparison tests (bracket). n = 10 for the MCAO+Vehicle group, n = 11 for the MCAO+3‐HKA group. n) Quantification of the contralateral CBF.

To determine the biological activity of 3‐HKA in regulating post‐stroke neurological deficits in vivo, we treated experimental animals with 3‐HKA. We used the rotarod test and the modified neurological severity score (mNSS) for initial dose screening and observed improved neurological recovery at 40 mg kg^−1^ and 80 mg kg^−1^ 3‐HKA. Considering a balance between efficacy and safety, we selected 40 mg kg^−1^ for all subsequent in vivo experiments (Figure S2a,b, Supporting Information). We then conducted a thorough assessment of sensorimotor functions prior to and up to 28 days following cerebral ischemic injury via a battery of behavioral measures (rotarod, mNSS, adhesive removal test and gait analysis). Compared with the sham group, the MCAO, MCAO+Vehicle, and MCAO+3‐HKA groups presented significant sensorimotor deficits in a series of behavioral tests, which were particularly noticeable in the first week following MCAO (**Figure** **2**a–c; Figure S2c, Supporting Information). No significant differences in neurological deficits were detected between the MCAO and MCAO+Vehicle groups at any time point (Figure 2a–c; Figure S2c, Supporting Information). Interestingly, 3‐HKA treatment improved long‐term neurological recovery compared with vehicle control treatment in all behavioral tests at most time points (Figure 2a–c; Figure S2c, Supporting Information). Compared with vehicle treatment, the rotarod test revealed that 3‐HKA treatment increased the duration of time spent on the rotarod at days 7, 14, 21 and 28 after stroke, indicating that 3‐HKA improved post‐stroke motor coordination and learning ability (Figure 2a). Consistently, the 3‐HKA‐treated mice presented lower neurological deficit scores than did the vehicle‐treated mice at 7, 14, 21 and 28 days after stroke (Figure S2c, Supporting Information). As expected, 3‐HKA‐treated mice exhibited faster reaction to touch and removal of tape from the forepaw (7–28 days after stroke) than vehicle‐treated mice did, indicating that 3‐HKA ameliorated post‐stroke sensory‐motor impairment (Figure 2b,c).

Using the Catwalk XT system to analyze the footprints of the animals on the sidewalk, we performed gait analysis to assess sensorimotor function and gait control indices. Compared with vehicle treatment, 3‐HKA treatment significantly increased the activity levels related to sensorimotor function, as reflected by a better average run speed (7‐14 days after stroke) (Figure 2d), a greater base of front and hind paw support (7‐14 days after stroke) (Figure 2e,f), a greater base of left paw support (3‐14 days after stroke) (Figure 2g), a significantly longer stride length (3‐14 days after stroke) (Figure 2h) and a larger pawprint area (7‐14 days after stroke) for the left front paw (LF) (Figure 2i). Additionally, 3‐HKA treatment significantly improved the maximum lateral deviation of the left front paw (LF) and the maximum longitudinal deviation of the left rear paw (LR) compared with those of the vehicle‐treated group at days 7 and 14 after stroke (Figure S2d,e, Supporting Information).

The infarct volume of each group at day 7 after stroke was shown in Figure 2j. The vehicle group displayed a significant cortical lesion. Consistent with the findings from neurobehavioral assessments, mice treated with 3‐HKA showed a marked reduction in infarct volume compared to those in the vehicle group (Figure 2j,k).

On the basis of the improved stroke outcomes in mice treated with 3‐HKA, we hypothesized that 3‐HKA could increase CBF during the recovery phase following stroke. To examine whether long‐term neurological recovery from ischemic stroke after 3‐HKA treatment is associated with CBF, we used laser speckle imaging to track spatiotemporal variations in CBF. There were no significant differences in the ipsilateral CBF between 3‐HKA‐treated mice and vehicle‐treated mice 3 days after MCAO. Interestingly, we observed that the ipsilateral CBF was significantly greater in the 3‐HKA‐treated group than in the vehicle‐treated group at days 7, 14, and 28 post‐stroke (Figure 2l,m). However, no significant differences were observed in the contralateral CBF between 3‐HKA‐treated mice and vehicle‐treated mice at any time point (Figure 2n). These results corroborated the above observations of improved long‐term neurological recovery in 3‐HKA‐treated stroke model mice and showed, for the first time, that 3‐HKA increased reconstruction of cerebral blood flow in injured ischemic tissue.

### 2.3 3‐HKA Promoted Angiogenesis and Attenuated Blood–Brain Barrier Permeability after Cerebral Ischemia

**Figure 3:**
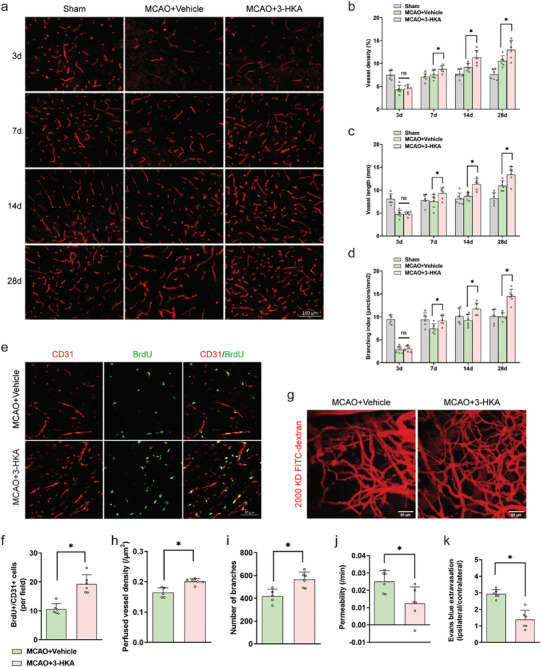
3‐HKA promoted angiogenesis and attenuated BBB permeability after cerebral ischemia. a) Representative images of CD31‐labeled blood vessels in the penumbral area at days 3, 7, 14, and 28 after cerebral ischemia. b) Quantification of vessel density. *P* = 0.034341 at 7 d, *P* =  0.004771 at 14 d, and *P* = 0.012615 at 28 d (MCAO+3‐HKA versus MCAO+Vehicle); one‐way ANOVA followed by LSD multiple comparison tests (individual time points). n = 6. c) Quantification of vessel length. *P* = 0.035448 at 7 d, *P* = 0.001163 at 14 d, and *P* = 0.008511 at 28 d (MCAO+3‐HKA versus MCAO+Vehicle); one‐way ANOVA followed by LSD multiple comparison tests (individual time points). n = 6. d) Quantification of the vessel branching index. *P* = 0.026134 at 7 d, *P* = 0.007609 at 14 d, and *P* = 0.00004 at 28 d (MCAO+3‐HKA versus MCAO+Vehicle); one‐way ANOVA followed by LSD multiple comparison tests (individual time points). n = 6. e,f) CD31 (red) and BrdU (green) double immunostaining was used to detect the newly formed microvessels in the peri‐infarct region at 14 d after MCAO. *P* = 0.000203 (MCAO+3‐HKA versus MCAO+Vehicle); Student's t test. n = 6. g–i) Representative two‐photon microscopy images of perfused vessels intravenously injected with FITC‐dextran (MW = 2000 kDa) in the peri‐infarct region at 14 days after MCAO g), with quantification of perfused vessel density h) and the number of branches i). Perfused vessel density, *P* = 0.001069; number of branches, *P* = 0.004484 (MCAO+3‐HKA (n = 6) versus MCAO+Vehicle (n = 5)); Student's t test. j) Quantification of the permeability of FITC‐dextran (MW = 40 kDa) at 14 days after cerebral ischemia. *P* = 0.021168 (MCAO+3‐HKA versus MCAO+Vehicle); Student's t test. n = 6. k) Quantification of Evans blue extravasation at 14 days after cerebral ischemia. *P* = 0.000113, MCAO+3‐HKA versus MCAO+Vehicle; Student's t test. n = 6.

It is generally believed that increased collateral flow may be the cause of an early increase in cerebral blood volume (CBV) in the ischemic hemisphere, whereas a surge in angiogenesis is thought to be responsible for the late‐phase increase in CBV.^[^ ^3^ ^]^ Therefore, we hypothesized that the increase in CBF recovery in the 3‐HKA group post‐stroke may be linked to improved vascular remodeling after stroke. We selected the peri‐infarct cortex as our focus area for studying revascularization because activated angiogenesis in this region is associated with prolonged survival in patients with ischemic stroke.^[^ ^3^, ^8^, ^21^ ^]^ As expected, the brain microvasculature at three days after stroke were significantly lower in both the vehicle‐treated group and the 3‐HKA‐treated group than in the sham groups (**Figure** **3**a). During the 28‐day recovery period, cerebral revascularization gradually increased in both the vehicle‐treated group and the 3‐HKA‐treated group (Figure 3a). However, the improvement was more significant in the mice treated with 3‐HKA (Figure 3a). Specifically, at 7 days, 14 days and 28 days after stroke, the vessel density, vessel length, branching index, and lacunarity in the peri‐infarct area of the 3‐HKA‐treated mice were generally greater than those in the vehicle‐treated mice (Figure 3a–d; Figure S3a, Supporting Information). Furthermore, we detected newly formed microvessels through CD31/BrdU double immunostaining. As shown in Figure 3e, there were more newly formed microvessels in the peri‐infarct area in the 3‐HKA‐treated group than in the vehicle‐treated group at 14 days after stroke (Figure 3e,f; Figure S3b, Supporting Information).

We subsequently evaluated the structural reshaping of the vasculature by using in vivo two‐photon microscopy for high‐resolution 3D reconstructions. Simultaneously, a vessel‐filling fluorescent dye, fluorescein isothiocyanate (FITC)‐dextran, was used to identify newly formed functional vessels. Notably, we discovered that 3‐HKA intervention triggered angiogenesis near the infarct site. Specifically, we found that the perfused vessel density and the number of branches in the ipsilateral cortex were notably greater in the 3‐HKA‐treated group than in the vehicle‐treated group 14 days after stroke (Figures 3g–i). Similar outcomes were observed as early as 7 days after stroke, which further suggests that 3‐HKA has biological activity in promoting angiogenesis after stroke (Figure S3c–e, Supporting Information).

The consequences of a stroke worsen rather than improve if the newly formed functional vessels are permeable. Therefore, we assessed the permeability of blood vessels through a series of in vivo and *ex vivo* measurements. Compared with vehicle‐treated mice, 3‐HKA‐treated mice presented considerable reductions in BBB permeability in the peri‐infarct areas at 14 days after stroke (Figure 3j). Moreover, the Evans blue extravasation rate in the peri‐infarct region was considerably lower in the 3‐HKA‐treated group than in the vehicle‐treated group at 14 days post‐stroke, suggesting that 3‐HKA could reduce vascular leakage after cerebral ischemia (Figure 3k). Overall, these results indicate that the promotion of neurological recovery after stroke by 3‐HKA intervention is associated with the enhancement of vascular remodeling in the penumbral region.

### 2.4 3‐HKA Triggered a Bolstering of A2‐Like Astrocytes While Suppressing A1‐Like Astrocytes After Stroke

**Figure 4:**
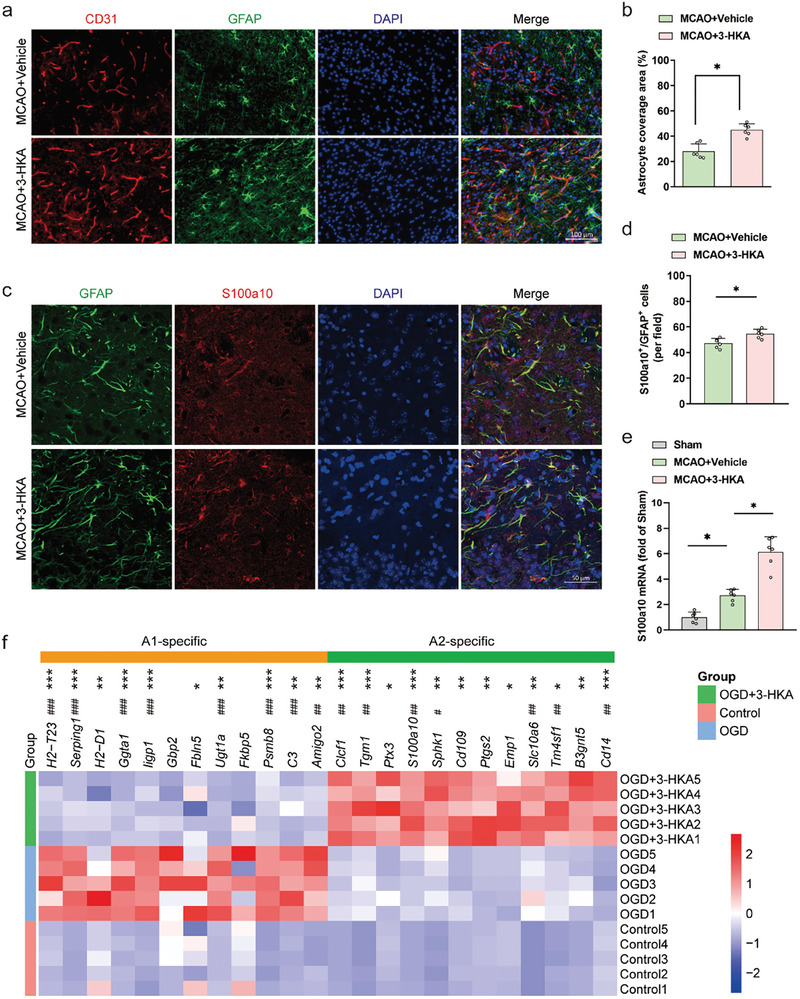
3‐HKA regulated the activation of A1/A2 subtype astrocytes after cerebral ischemia. a) CD31 (red, endothelial cell marker) and GFAP (green, astrocyte marker) double immunostaining was used to determine astrocyte coverage in the peri‐infarct region at 7 days post‐stroke. b) Quantification of the astrocyte coverage ratio. *P* = 0.000277 (MCAO+3‐HKA versus MCAO+Vehicle); Student's t test. n = 6. c) GFAP (green) and S100a10 (red) double immunostaining was used to identify A2‐like astrocytes in the peri‐infarct region at 7 days post‐stroke. d) Quantification of A2‐like astrocytes. *P* = 0.005518 (MCAO+3‐HKA versus MCAO+Vehicle); Student's t test. n = 6. e) mRNA expression of *S100a10* at 7 days post‐stroke. *P* = 0.001706 (MCAO+Vehicle versus Sham); *P* = 0.000002 (MCAO+3‐HKA versus MCAO+Vehicle); one‐way ANOVA followed by LSD multiple comparison tests. n = 6. f) Heatmap of the relative mRNA levels of target genes in A1‐like astrocytes and A2‐like astrocytes. ^#^ *P* < 0.05, *^##^P* < 0.01, *^###^P* < 0.001 (OGD versus Control); *P < 0.05, **P < 0.01, ***P < 0.001 (OGD+3‐HKA versus OGD). n = 5.

Astrocytes, as constituent cells of the BBB, participate in many vascular remodeling processes and are considered important regulators of vascular remodeling.^[^ ^8^ ^]^ Recent studies have shown that ischemia can induce two different types of reactive astrocytes, namely, A1‐like (neurotoxic) astrocytes, which are characterized by complement C3, and A2‐like (neuroprotective) astrocytes, which are characterized by S100a10.^[^ ^22^, ^23^ ^]^ Our results revealed that, compared with vehicle, 3‐HKA significantly increased the area of astrocyte coverage around microvessels 7 days after stroke (**Figure** **4**a,b). We subsequently assessed A2‐like (S100a10^+^/GFAP^+^) astrocytes in the peri‐infarct region at 7 days post‐stroke and discovered a noteworthy increase in the 3‐HKA‐treated group compared with the vehicle‐treated group (Figure 4c,d). In support of this, we found that the mRNA level of *S100a10* was significantly greater in 3‐HKA‐treated mice than in vehicle‐treated mice (Figure 4e).

Primary astrocytes were subsequently isolated and cultured in vitro to verify the effects of 3‐HKA on the development of A1‐like and A2‐like astrocytes after oxygen‒glucose deprivation (OGD). After OGD, the expression of genes associated with both “A1‐like” or “A2‐like” astrocytes was altered. As shown in Figure 4f, the mRNA expression of 12 A1‐like astrocyte‐specific genes was significantly downregulated, and that of 12 genes specific to A2‐like astrocytes was upregulated, in the 3‐HKA‐treated group after OGD (Figure 4f). Ultimately, our findings demonstrated that 3‐HKA promoted the formation of A2‐like astrocytes and hindered the formation of A1‐like astrocytes following stroke.

### 2.5 3‐HKA Regulated Astrocyte A1/A2 Transition by Inhibiting the Activation of the AIM2 Inflammasome During Stroke

**Figure 5:**
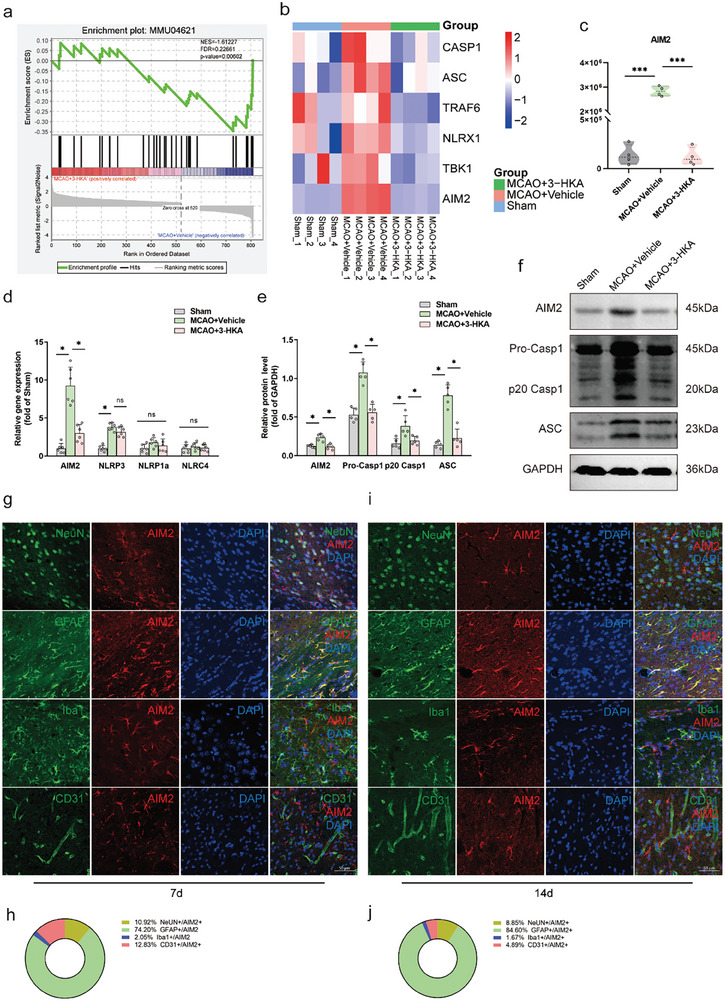
3‐HKA attenuated AIM2 inflammasome activation after stroke. a) PSEA of differentially expressed proteins in the NOD‐like receptor signaling pathway based on the proteomics data. NES = ‐1.61227, FDR = 0.22861, p‐value = 0.00602. NES: net enrichment score; FDR: false discovery rate. n = 4. b) Heatmap of representative proteins in the NOD‐like receptor signaling pathway from the proteomics data. n = 4. c) Violin plot showing the relative differences in the levels of AIM2 in the proteomics data. ***P < 0.001, n = 4. d) Relative gene expression of *AIM2*, *NLRP3*, *NLRP1a*, and *NLRC4*. *AIM2*: *P* = 0.000763 (MCAO+Vehicle versus Sham); *P* = 0.002107 (MCAO+3‐HKA versus MCAO+Vehicle); one‐way ANOVA followed by Dunnett's multiple comparison tests. *NLRP3*: *P* < 0.001 (MCAO+Vehicle versus Sham); one‐way ANOVA followed by LSD multiple comparison tests. n = 6. e,f) The protein levels of AIM2, pro‐caspase‐1, p20 caspase‐1 and ASC in each group. AIM2, *P* = 0.000265 (MCAO+Vehicle versus Sham), *P* = 0.000133 (MCAO+3‐HKA versus MCAO+Vehicle); pro‐caspase‐1, *P* = 0.000005 (MCAO+Vehicle versus Sham), *P* = 0.000010 (MCAO+3‐HKA versus MCAO+Vehicle); p20 caspase‐1, *P* = 0.002106 (MCAO+Vehicle versus Sham), *P* = 0.006587 (MCAO+3‐HKA versus MCAO+Vehicle); ASC, *P* = 0.000001 (MCAO+Vehicle versus Sham), *P* = 0.000003 (MCAO+3‐HKA versus MCAO+Vehicle); one‐way ANOVA followed by LSD multiple comparison tests. n = 5. g,h) Representative immunostaining images and quantification graphs showing NeuN+/AIM2+, GFAP+/AIM2+, Iba1+/AIM2+ or CD31+/AIM2+ cells in the ischemic penumbra on days 7 after cerebral ischemia. AIM2 (red), NeuN (green, neuronal marker), GFAP (green), Iba‐1 (green, microglial marker) or CD31 (green). i,j) Representative immunostaining images and quantification of NeuN+/AIM2+, GFAP+/AIM2+, Iba1+/AIM2+ or CD31+/AIM2+ cells in the penumbral area on day 14 after cerebral ischemia.

To explore the molecular mechanism by which 3‐HKA regulates the astrocyte A1/A2 transition after stroke, we subsequently performed proteomic data analysis of mice treated with 3‐HKA or vehicle. There were 591 differentially expressed proteins (DEPs) in the vehicle‐treated group compared with the sham group, including 255 upregulated proteins and 336 downregulated proteins (Figure S4a–c, Supporting Information). In addition, when the 3‐HKA‐treated group was compared with the vehicle‐treated group, proteomic data analysis revealed 809 DEPs, comprising 520 upregulated and 289 downregulated proteins (Figure S4a–c, Supporting Information). Furthermore, the Nod‐like receptor (NLR) signaling pathway was the top‐ranked pathway enriched by Protein Set Enrichment Analysis (PSEA) when comparing the 3‐HKA‐treated group to the vehicle‐treated group (**Figure** **5**a), with notable changes observed in the proteins Caspase 1, ASC, TRAF6, NLRX1, TBK1, and AIM2 (Figure 5b). Compared with the sham group, the AIM2 protein levels were increased following stroke, and the increased AIM2 protein levels were reversed by 3‐HKA treatment (Figure 5c).

To further verify the mechanism by which 3‐HKA inhibits the activation of the AIM2 inflammasome after stroke, we also examined the mRNA expression of other Nod‐like receptors, such as *NLRP3*, *NLRP1a* and *NLRC4*. Our results demonstrated that, compared with those in the sham group, the mRNA levels of *AIM2* and *NLRP3*, but not those of *NLRP1a* and *NLRC4*, were significantly elevated following stroke. 3‐HKA treatment resulted in a significant decrease in the mRNA level of *AIM2* following stroke but had no obvious effect on *NLRP3* (Figure 5d). The expression of essential molecules of the AIM2 inflammasome, including AIM2, pro‐caspase‐1, p20 caspase‐1, and ASC was evaluated via Western blotting. We found that the expression of AIM2, pro‐caspase‐1, p20 caspase‐1, and ASC increased after cerebral ischemia, suggesting the activation of the AIM2 inflammasome, and this increase was reversed upon 3‐HKA treatment (Figure 5e,f). Next, we performed co‐localization analysis of AIM2 with NeuN (a neuronal marker), GFAP (an astrocyte marker), Iba1 (a microglial marker), and CD31 (an endothelial cell marker) in the penumbral area at days 7 and 14 after cerebral ischemia. We found that AIM2 was co‐localized mainly with astrocytes, a few neurons and endothelial cells, with almost no co‐localization with microglia at days 7 (Figure 5g,h) and 14 (Figures 5I,j) after stroke.

**Figure 6:**
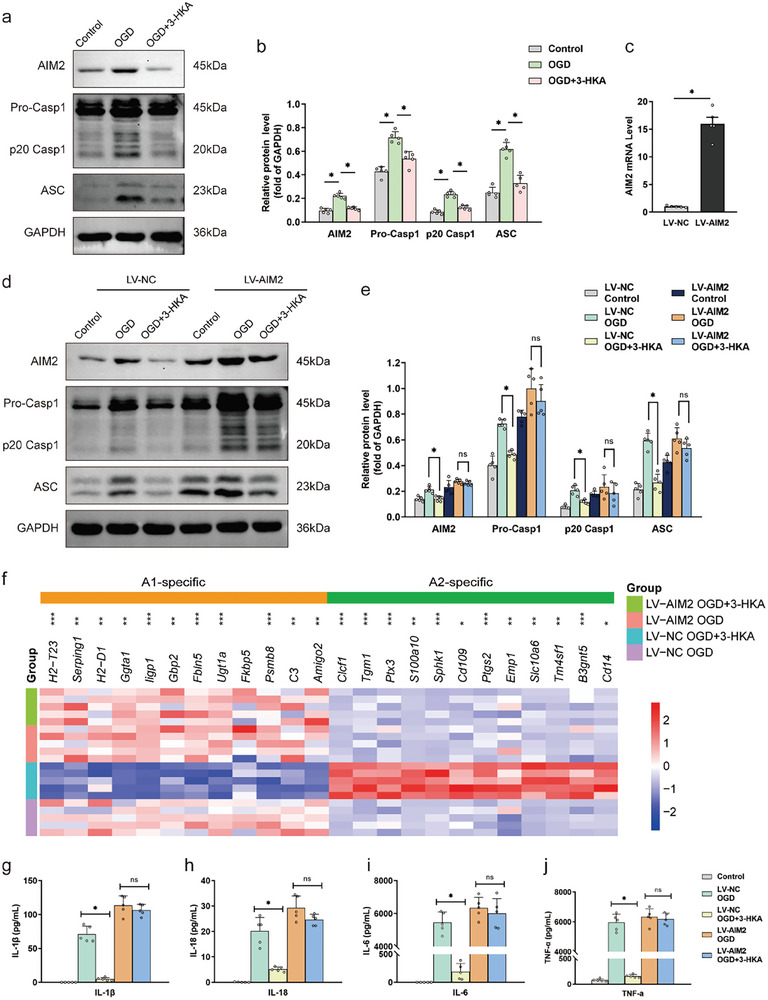
3‐HKA regulated the astrocyte A1/A2 transition by inhibiting the activation of the AIM2 inflammasome after stroke. a,b) Protein levels of AIM2, pro‐caspase‐1, p20 caspase‐1 and ASC in each group of primary astrocytes. AIM2, *P* < 0.001 (OGD versus Control), *P* = 0.000002 (OGD+3‐HKA versus OGD); pro‐caspase‐1: *P* = 0.000001 (OGD versus Control), *P* = 0.000132 (OGD+3‐HKA versus OGD); p20 caspase‐1: *P* < 0.001 (OGD versus Control), *P* = 0.000002 (OGD+3‐HKA versus OGD); ASC: *P* < 0.001 (OGD versus Control), *P* = 0.000003 (OGD+3‐HKA versus OGD); one‐way ANOVA followed by LSD multiple comparison tests. n = 5. c) Relative gene expression of *AIM2* after LV‐AIM2 treatment. *P* = 0.000207 (LV‐AIM2 versus LV‐NC) by Student's t‐test. n = 5. d,e) The protein levels of AIM2, pro‐caspase‐1, p20 caspase‐1 and ASC in each group in primary astrocytes. AIM2, *P* = 0.017599 (LV‐NC OGD+3‐HKA versus LV‐NC OGD); pro‐caspase‐1, *P* = 0.00004 (LV‐NC OGD+3‐HKA versus LV‐NC OGD); one‐way ANOVA followed by Dunnett's multiple comparison tests; p20 caspase‐1, *P =* 0.031 (LV‐NC OGD+3‐HKA versus LV‐NC OGD); Kruskal‒Wallis test followed by Dunn's post hoc analysis. ASC, *P* < 0.001 (LV‐NC OGD+3‐HKA versus LV‐NC OGD); one‐way ANOVA followed by LSD multiple comparison tests. n = 5. f) Heatmap of the relative mRNA levels of target genes in A1‐like astrocytes and A2‐like astrocytes. *P < 0.05, **P < 0.01, ***P < 0.001, LV‐NC OGD+3‐HKA versus LV‐NC OGD, n = 5. g–j) The concentrations of IL‐1β, IL‐18, IL‐6, and TNF‐α in the supernatant of primary astrocytes. IL‐1β, *P* = 0.001079 (LV‐NC OGD+3‐HKA versus LV‐NC OGD); IL‐18, *P* = 0.018370 (LV‐NC OGD+3‐HKA versus LV‐NC OGD); IL‐6, *P* < 0.001 (LV‐NC OGD+3‐HKA versus LV‐NC OGD); TNF‐α, *P* = 0.000118 (LV‐NC OGD+3‐HKA versus LV‐NC OGD); one‐way ANOVA followed by Dunnett's multiple comparison tests. n = 5.

We further assessed whether 3‐HKA regulates A1/A2 astrocyte transition by inhibiting the activation of the AIM2 inflammasome. The minimum effective dose of 3‐HKA in primary astrocytes was 10 µM, as determined by a cell viability assay (Figure S5, Supporting Information). The expression of essential molecules of the AIM2 inflammasome, including AIM2, pro‐caspase‐1, p20 caspase‐1, and ASC was evaluated via Western blotting. We found that OGD increased the expression of AIM2, pro‐caspase‐1, p20 caspase‐1, and ASC in primary astrocytes, but this increase in expression was abolished by 3‐HKA treatment (**Figure** **6**a,b). We subsequently overexpressed AIM2 in primary astrocytes via lentiviral infection and confirmed successful overexpression of AIM2 at the mRNA level (Figure 6c). AIM2 overexpression completely abrogated the inhibitory effect of 3‐HKA treatment on AIM2 inflammasome activation (Figure 6d,e). Moreover, 3‐HKA promoted A2‐like astrocyte development and inhibited A1‐like astrocyte development after OGD, but this protective activity of 3‐HKA was abolished when AIM2 was overexpressed (Figure 6f). As expected, overexpression of AIM2 eliminated the decreases in the levels of cytokines, such as IL‐1β, IL‐18, IL‐6 and TNF‐α, in the supernatants of primary astrocytes induced by 3‐HKA treatment (Figure 6g–j). Collectively, these findings showed that 3‐HKA influences the transition of astrocytes from a neurotoxic phenotype to a neuroprotective phenotype by preventing the activation of the AIM2 inflammasome, thereby limiting the inflammatory milieu.

### 2.6 3‐HKA Promoted Angiogenesis after OGD In Vitro via Regulation of the Astrocyte A1/A2 Transition by Impeding AIM2 Inflammasome Activation

**Figure 7:**
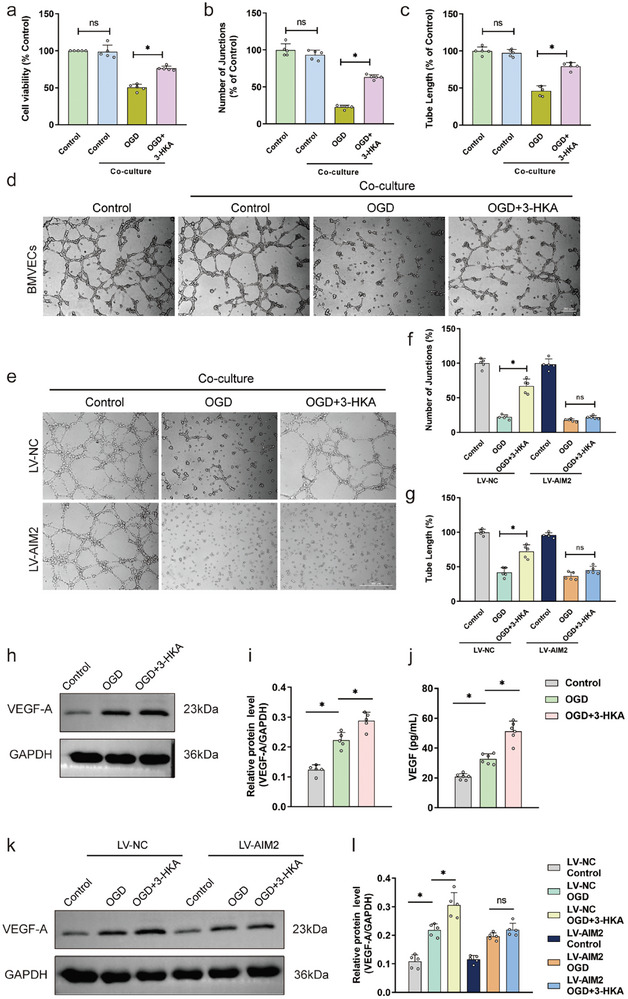
3‐HKA facilitates angiogenesis in vitro via regulation of the astrocyte A1/A2 transition by inhibiting activation of the AIM2 inflammasome. a) Viability of BMVECs co‐cultured endothelial cells and astrocytes. *P* = 0.000088 (Co‐OGD+3‐HKA versus Co‐OGD); one‐way ANOVA followed by Dunnett's multiple comparison tests. n = 5. b) Number of junctions in the capillary tube formation assay in co‐culture of endothelial cells and astrocytes. *P* < 0.001 (Co‐OGD+3‐HKA versus Co‐OGD); one‐way ANOVA followed by LSD multiple comparison test. n = 5. c) Tube length in the capillary tube formation assay of co‐cultured endothelial cells. *P* < 0.001 (Co‐OGD+3‐HKA versus Co‐OGD); one‐way ANOVA followed by LSD multiple comparison test. n = 5. d) Representative images of the capillary tube formation assay. e) Representative images of the capillary tube formation assay in co‐culture of endothelial cells and astrocytes. f) Number of junctions in the capillary tube formation assay in co‐culture of endothelial cells and astrocytes. *P* = 0.002736 (LV‐NC OGD+3‐HKA versus LV‐NC OGD); one‐way ANOVA followed by Dunnett's multiple comparison tests. n = 5. g) Tube length in the capillary tube formation assay in co‐culture of endothelial cells and astrocytes. *P* = 0.006935 (LV‐NC OGD+3‐HKA versus LV‐NC OGD); one‐way ANOVA followed by Dunnett's multiple comparison tests. n = 5. h,i) The protein levels of VEGF‐A in primary astrocytes. *P* = 0.000033 (OGD versus Control), *P* = 0.001176 (OGD+3‐HKA versus OGD); one‐way ANOVA followed by LSD multiple comparison tests. n = 5. j) The concentrations of VEGF in the supernatant of primary astrocytes. *P* = 0.000346 (OGD versus Control), *P* = 0.000003 (OGD+3‐HKA versus OGD); one‐way ANOVA followed by LSD multiple comparison tests. n = 6. k,l) The protein levels of VEGF‐A in primary astrocytes. *P* < 0.001 (LV‐NC OGD versus LV‐NC Control), *P* = 0.000014 (LV‐NC OGD+3‐HKA versus LV‐NC OGD); one‐way ANOVA followed by LSD multiple comparison tests. n = 5.

We further investigated the impact of 3‐HKA on angiogenesis via cell viability and capillary tube formation assays of primary mouse brain microvascular endothelial cells (BMVECs). Contrary to our expectations, 3‐HKA did not increase the proliferation (Figure S6a, Supporting Information) or capillary tube formation (Figure S6b–d, Supporting Information) of endothelial cells after OGD, suggesting that 3‐HKA does not directly stimulate angiogenesis in vitro, which contradicts our in vivo findings. On the basis of this finding and the above observation that 3‐HKA promotes the neuroprotective A2‐like astrocyte phenotype, we speculated that 3‐HKA may promote angiogenesis by regulating the A1/A2 astrocyte transition. To test this hypothesis, we co‐cultured endothelial cells and astrocytes (Figure S8, Supporting Information). As predicted by our hypothesis, cell viability assays revealed that OGD decreased endothelial cell proliferation in the co‐culture of endothelial cells and astrocytes, whereas 3‐HKA promoted endothelial cell proliferation (**Figure** **7**a). Moreover, tube formation assays following endothelial cell/astrocyte co‐culture demonstrated that OGD caused a reduction in the number of junctions and tube length that could be reversed by 3‐HKA (Figure 7b–d). To further confirm that 3‐HKA exerts its pro‐angiogenic effects by regulating AIM2 inflammasome activation in astrocytes, AIM2 was overexpressed in astrocytes via lentiviral infection. Notably, overexpression of AIM2 completely abrogated the 3‐HKA‐induced increase in angiogenesis, as evidenced by decreases in the number of junctions and in tube length (Figure 7e–g).

Vascular endothelial growth factor (VEGF) is a key driver of angiogenesis, and astrocyte‐derived VEGF plays a pivotal role in vascular remodeling.^[^ ^8^, ^24^, ^25^, ^26^ ^]^ We investigated whether 3‐HKA facilitated astrocyte‐derived VEGF by suppressing AIM2 inflammasome activation. Our findings revealed that OGD significantly increased VEGF‐A protein expression in primary astrocytes, and this increase was further augmented by 3‐HKA treatment (Figure 7h,i). Correspondingly, VEGF secretion in the supernatant of primary astrocytes increased after OGD, with 3‐HKA treatment further amplifying this effect (Figure 7j). However, overexpression of AIM2 negated the stimulatory effect of 3‐HKA on astrocyte‐derived VEGF, suggesting that 3‐HKA may enhance astrocyte‐derived VEGF by impeding AIM2 inflammasome activation (Figure 7k,l). Taken together, these results confirmed that 3‐HKA inhibited AIM2 inflammasome activation, promoting A2‐like polarization of astrocytes and resulting in increased astrocyte‐derived VEGF, which facilitated angiogenesis after OGD.

### 2.7 3‐HKA Increased Vascular Remodeling Post‐Stroke In Vivo by Hindering the Activation of AIM2 Inflammasomes

**Figure 8:**
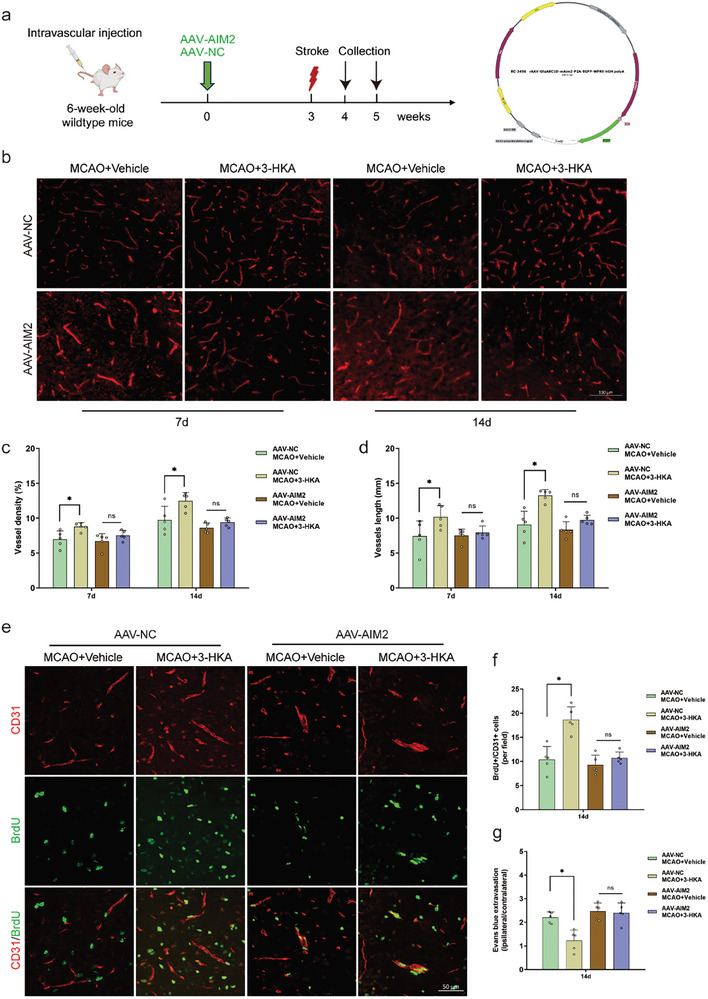
3‐HKA increased vascular remodeling post‐stroke in vivo via regulation of the astrocyte A1/A2 transition by inhibiting activation of the AIM2 inflammasome. a) To induce overexpression of the AIM2 gene in astrocytes, we constructed an AAV‐AIM2 viral vector. Schematic and timeline of the experiments used to assess vascular remodeling in mice receiving intravascular injection of AAV‐NC or AAV‐AIM2 (left). Genetic representation of AAV‐AIM2, where colors and arrows indicate the represented genes and their orientation (right). b) Representative images of vessels in the penumbral area on days 7 and 14 after cerebral ischemia. c) Quantification of vessel density. MCAO 7 d, *P* = 0.007488 (AAV‐NC MCAO+3‐HKA versus AAV‐NC MCAO+Vehicle); MCAO 14 d, *P* = 0.003374 (AAV‐NC MCAO+3‐HKA versus AAV‐NC MCAO+Vehicle); one‐way ANOVA followed by LSD multiple comparison tests. n = 5. d) Quantification of vessel length. MCAO 7 d, *P* = 0.011736 (AAV‐NC MCAO+3‐HKA versus AAV‐NC MCAO+Vehicle); MCAO 14 d, *P* = 0.000068 (AAV‐NC MCAO+3‐HKA versus AAV‐NC MCAO+Vehicle); one‐way ANOVA followed by LSD multiple comparison tests. n = 5. e,f) Quantification of CD31^+^/BrdU^+^ cells in the peri‐infarct region at 14 d after MCAO. *P* = 0.000025 (AAV‐NC MCAO+3‐HKA versus AAV‐NC MCAO+Vehicle); Student's t test. n = 5. g) Quantification of Evans blue extravasation at 14 days after cerebral ischemia. *P* = 0.000671 (AAV‐NC MCAO+3‐HKA versus AAV‐NC MCAO+Vehicle); one‐way ANOVA followed by LSD multiple comparison tests. n = 5.

To further corroborate these findings in vivo, we intravascularly injected an astrocyte‐targeting adeno‐associated virus expressing AIM2 (AAV‐AIM2) to overexpress AIM2 throughout the brain (**Figure** **8**a). To examine whether AAV‐AIM2 mediated gene expression was restricted to astrocytes, EGFP (AAV‐AIM2) and GFAP signals were imaged as previously described.^[^ ^27^ ^]^ Our findings demonstrated the significant co‐localization of EGFP and GFAP signals in coronal sections of the brain, indicating that AAV‐AIM2 effectively targeted astrocytes (Figure S7a,b, Supporting Information). As previously indicated, vessel density, vessel length, the branching index, and lacunarity were generally greater in the peri‐infarct area of 3‐HKA‐treated mice than in that of vehicle‐treated mice at 7 and 14 days after stroke. However, overexpression of AIM2, which targets astrocytes, counteracted the proangiogenic effect of 3‐HKA (Figure 8b–d; Figure S7c,d, Supporting Information). Next, we detected newly formed microvessels in the peri‐infarct area at 14 days post‐stroke through CD31/BrdU double immunostaining. At 14 days after stroke, the number of newly formed microvessels was increased in the 3‐HKA‐treated group, but this increase was suppressed by AIM2 overexpression (Figure 8e,f). To assess BBB breakdown, we measured the Evans blue extravasation rate. Specifically, 14 days after stroke, damage to the BBB in the 3‐HKA‐treated group was greater than that in the vehicle‐treated group, and overexpression of AIM2 offset this protective effect (Figure 8g). Collectively, these results suggested that 3‐HKA promotes vascular remodeling after stroke by transforming A1‐like into A2‐like astrocytes through impeding AIM2 inflammasome activation.

## 3 Discussion

Ischemic stroke, the most common cerebrovascular disease and the leading cause of permanent disability worldwide, is a substantial burden on society and families, yet there are few treatments available to improve long‐term recovery. Here, we revealed that 3‐HKA, a tryptophan metabolite, was significantly downregulated in ischemic stroke patients and mice. Supplementation with 3‐HKA improved long‐term neurological recovery, reduced cerebral infarct volume, and increased ipsilateral CBF after MCAO. 3‐HKA also promoted angiogenesis, functional blood vessel formation, and BBB repair, thereby improving post‐stroke neurological recovery. Intriguingly, 3‐HKA inhibited A1‐like (neurotoxic) astrocyte activation but promoted A2‐like (neuroprotective) astrocyte polarization. Mechanistically, we found that 3‐HKA facilitated endothelial cell proliferation by hindering the activation of AIM2 inflammasomes in astrocytes, reducing inflammatory cytokine release and increasing astrocyte‐derived VEGF. AIM2 overexpression in astrocytes abrogated 3‐HKA‐driven vascular remodeling in vitro and in vivo, indicating that 3‐HKA may regulate astrocyte‐mediated vascular remodeling by impeding AIM2 inflammasome activation. Importantly, our findings identify 3‐HKA as a critical regulator of vascular remodeling after cerebral ischemia via the regulation of the astrocyte A1/A2 transition by impeding AIM2 inflammasome activation and suggest that supplementation with 3‐HKA may be efficient in the therapeutic management of stroke.

Tryptophan can be metabolized by mammals and their gut bacteria mainly through the kynurenine, serotonin, and indole pathways, to produce a variety of bioactive compounds.^[^ ^15^, ^16^, ^28^, ^29^, ^30^, ^31^ ^]^ The biological activity of certain tryptophan metabolites, including as neurotoxins (quinolinic acid), hormones (melatonin), and neurotransmitters (serotonin), is well established in mammals; however, the functional importance of many other tryptophan metabolites is still completely unknown.^[^ ^20^, ^32^ ^]^ According to clinical research, ischemic stroke patients have an elevated kynurenine/tryptophan ratio and a significantly decreased 3‐hydroxyanthranilic acid/anthranilic acid ratio, which is strongly associated with infarct volume. Thus, blocking the kynurenine metabolic pathway could reduce patient morbidity and death.^[^ ^18^ ^]^ In addition, screens of the plasma metabolome of patients with transient ischemic attack (TIA) performed to identify metabolites with differential expression identified five metabolites (atDR, 20‐carboxyleukotriene B4, prostaglandin B2, cortisol, and 9‐KODE) that are significantly upregulated after TIA and can prevent neurological deficits in MCAO mice.^[^ ^28^ ^]^ In the present study, analysis of untargeted metabolomic data from acute stroke patients revealed that the tryptophan metabolite 3‐HKA was significantly decreased, and we successfully verified this finding via tryptophan pathway‐targeted metabolomic analysis of MCAO mice. Does 3‐HKA contribute to stroke biology? To answer this question, we exogenously administered 3‐HKA to stroke model mice and found that 3‐HKA can promote long‐term neurological recovery after stroke, thus providing new ideas for stroke treatment.

Angiogenesis serves as a vital repair mechanism following cerebral ischemia, promoting vascular regeneration and functional recovery. Angiogenesis refers to the formation of new vascular structures via sprouting from pre‐existing blood vessels. New blood vessels can increase tissue perfusion around the ischemic area and release a series of neurotrophic factors, such as VEGF and BDNF, that are conducive to neuroregeneration and neuroplasticity.^[^ ^3^, ^8^, ^33^ ^]^ Nevertheless, this type of angiogenesis is somewhat self‐limited, and the BBB of new blood vessels is not yet complete. The destruction of BBB integrity leads to vasogenic brain tissue edema and inflammatory cell migration and infiltration into ischemic brain tissue, further aggravating brain tissue damage.^[^ ^2^, ^34^, ^35^ ^]^ Vascular remodeling is therefore essential for preserving a stable brain microenvironment and improving the recovery of neurological function following stroke. Angiogenesis occurs in the ischemic penumbra as early as 12 h after experimental cerebral ischemia, is most obvious at ≈7–14 days, and may last up to 28 days, consistent with our experimental results.^[^ ^6^, ^8^, ^21^, ^24^, ^36^ ^]^ Our results illustrate that 3‐HKA can promote vascular remodeling of the ischemic peripheral cortex at 7 days after ischemia and persisted for up to 28 days, which aids in the restoration of CBF in the ischemic cerebral hemisphere; in particular, previous work has shown that active angiogenesis in the ischemic penumbra is associated with a higher survival rate after ischemic stroke.^[^ ^37^, ^38^ ^]^ According to our research findings, 3‐HKA promotes vascular remodeling to increase CBF and improve long‐term neurological recovery following stroke.

Astrocytes are common cells in the central nervous system (CNS) that perform a variety of homeostatic maintenance tasks, including providing trophic support for neurons, promoting synapse formation, and promoting BBB formation and function.^[^ ^22^, ^35^, ^36^, ^39^, ^40^ ^]^ “Reactive astrogliosis” is the term used to describe the significant change that occurs in astrocytes following brain damage. Research has revealed that two distinct subtypes of reactive astrocytes, known as neurotoxic “A1” and neuroprotective “A2” astrocytes, are affected by neuroinflammation and ischemia. A1‐ phenotype astrocytes exhibit substantial upregulation of the expression of many destructive classic complement cascade genes, whereas A2‐phenotype astrocytes exhibit upregulation of the expression of many neurotrophic factors.^[^ ^22^, ^23^, ^41^, ^42^, ^43^, ^44^ ^]^ However, some scholars contend that this binary separation is insufficiently complex to encompass the transcriptomes of all astrocytes. The terms “A1‐like” and “A2‐like” are more relevant because the astrocyte phenotype should be determined by a combination of genetic markers and functional readouts, such as the 12 astrocyte‐specific genes used in the study.^[^ ^43^ ^]^ Moreover, analysis of our data revealed that after stroke, astrocytes exhibited upregulation of not only the expression of “A2‐like” genes but also the expression of “A1‐like” genes, which supports and extends the findings of previous studies.^[^ ^22^, ^45^ ^]^ In addition, studies have shown that reducing A1‐like astrocyte formation or promoting the conversion of A1‐like astrocytes to A2‐like astrocytes improves synaptic plasticity in neurodegenerative diseases, including AD, Huntington's disease (HD), Parkinson's disease (PD), and amyotrophic lateral sclerosis (ALS).^[^ ^22^, ^42^, ^46^ ^]^ Other studies have shown that ablation of reactive astrocytes after stroke adversely affects angiogenesis and BBB leakage, but the phenotypes of these astrocytes have not been characterized.^[^ ^35^ ^]^ Our data suggest that promoting the transformation of A1‐like astrocytes to A2‐like astrocytes is beneficial for vascular remodeling, positioning A2‐like astrocytes as the major regulators of vascular remodeling after cerebral ischemia.

A thorough understanding of the mechanism underlying the development of A2‐like astrocytes is essential because these cells have neuroprotective benefits such as the ability to support neurogenesis and synaptic plasticity, as well as the capacity to promote vascular remodeling, as discussed herein. A previous study revealed that after cerebral ischemia, astrocyte KLF4 promotes the transformation of A1‐like astrocytes into A2‐like astrocytes by regulating the expression of NF‐κB.^[^ ^23^ ^]^ Some researchers have also reported that astrocyte glycogen mobilization after cerebral ischemia inhibits A1‐like astrocytes and promotes A2‐like astrocytes, among which reactive oxygen species (ROS)‐mediated NF‐κB suppression and STAT3 activation are important routes for glycogen mobilization‐induced neuroprotection.^[^ ^43^ ^]^ Our data revealed that 3‐HKA can promote the transformation of A1‐like into A2‐like astrocytes by inhibiting the activation of the AIM2 inflammasome, a multiprotein complex composed of AIM2, ASC, and caspase‐1 that is assembled upon AIM2 activation.^[^ ^47^, ^48^, ^49^, ^50^, ^51^ ^]^ Bacterial double‐stranded DNA (dsDNA) is released into the cytoplasm of infected cells when the host is infected. AIM2, a cytoplasmic pattern recognition receptor (PRR), recognizes the dsDNA of the pathogen and triggers the oligomerization of sensors, initiating the recruitment of ASC and activating caspase‐1. Activated caspase‐1 cleaves pro‐IL‐1β and pro‐IL‐18 to produce mature, biologically active IL‐1β and IL‐18, which are secreted into the extracellular environment to trigger inflammatory responses.^[^ ^48^, ^52^, ^53^, ^54^, ^55^, ^56^ ^]^

In this study, we examined the expression patterns of the AIM2 inflammasome and other cytoplasmic PRRs, including NLRP1a, NLRP3, and NLRC4, in mice after stroke and found that AIM2 and NLRP3 were activated 7 days after cerebral ischemia. After 3‐HKA intervention, the activation of AIM2 but not NLRP3 was inhibited, indicating that 3‐HKA exerts its effects specifically through AIM2; thus, the AIM2 inflammasome was selected for further investigation. It remains controversial which cell types are impacted by AIM2 inflammasome activation in the CNS after stroke. Some studies using immunofluorescence analyses have shown that in transient MCAO models, AIM2 co‐localized primarily with microglia and endothelial cells.^[^ ^57^, ^58^ ^]^ In contrast to these other studies, our findings demonstrated that AIM2 was expressed primarily in astrocytes and, to a lesser extent, in neuronal cells, microglia, and endothelial cells in the ischemic penumbra at 7 and 14 days after stroke caused by distal middle cerebral artery occlusion, which is a crucial time point for vascular remodeling. The difference in AIM2 localization among studies may be related to differences in the time points evaluated and the stroke models used. However, this finding, along with the fact that 3‐HKA could not directly promote endothelial cell tube formation in vitro, shows that 3‐HKA facilitates vascular remodeling through astrocytes. VEGF is a potent secreted signaling protein that plays a crucial role in vascular homeostasis, particularly in angiogenesis and blood‐brain barrier repair.^[^ ^3^, ^24^, ^59^, ^60^ ^]^ In vitro studies have demonstrated that VEGF is essential for the proliferation and migration of endothelial cells.^[^ ^8^ ^]^ Our data revealed that following OGD, there was an increase in astrocyte‐derived VEGF, which was significantly augmented by 3‐HKA treatment. Notably, overexpression of AIM2 negated the positive effects of 3‐HKA, suggesting that the mechanism by which 3‐HKA promotes vascular remodeling may involve the inhibition of AIM2 inflammasome activation, thereby facilitating increased astrocyte‐derived VEGF. Research has shown that repetitive transcranial magnetic stimulation (rTMS) can enhance the polarization of A2 astrocytes, leading to elevated astrocyte‐derived VEGF levels; however, the specific mechanisms remain unclear.^[^ ^39^ ^]^ Furthermore, in retinal vascular development models, astrocyte‐derived VEGF has been shown to promote the radial migration of endothelial cells.^[^ ^26^ ^]^ The protective effect of astrocyte‐derived VEGF on blood‐brain barrier integrity has also been confirmed in mouse models of Parkinson's disease.^[^ ^61^ ^]^ Our research extends previous research by demonstrating that the activation of the AIM2 inflammasome within astrocytes plays a crucial role in regulating astrocyte‐derived VEGF. Collectively, these results confirmed that 3‐HKA inhibited AIM2 inflammasome activation, promoting A2‐like polarization of astrocytes and resulting in increased astrocyte‐derived VEGF, which facilitated vascular remodeling after stroke.

This study has several limitations that should be considered. First, it remains to be seen whether 3‐HKA could promote neurological recovery after stroke through other mechanisms. In addition, our research was limited to rodent models, necessitating further preclinical translational research on 3‐HKA in non‐human primates.

In summary, this study revealed a reduction in the levels of the tryptophan metabolite 3‐HKA after stroke, and the administration of 3‐HKA promoted vascular remodeling after experimental ischemic stroke by regulating A1/A2 reactive astrocytes. These results indicate that 3‐HKA holds promise as a therapeutic target for promoting neurological recovery after stroke. Therefore, 3‐HKA could offer new avenues for developing innovative restorative treatments for ischemic stroke.

## 4 Experimental Section

### Patients and Sampling

Blood samples were obtained from 46 patients with AIS (25 males, 21 females; age 60.72 ± 1.45 years) and 35 age‐ and sex‐matched healthy controls (18 males, 17 females; age 57.29 ± 1.69 years). The study received approval from the Medical Ethics Committee of the Second Hospital of Hebei Medical University (Approval No. 2024‐R163, Hebei, China), and all participants provided informed consent. AIS patients were enrolled within 24 h of symptom onset, adhering to the 2018 Chinese Guidelines for Acute Ischemic Stroke. To ensure consistency, participants were not using probiotics or antibiotics in the month prior to the study, and those with a history of alcohol or substance dependence, or who were pregnant or lactating, were excluded.

After collection, blood samples were allowed to stand at room temperature for 30 min before being centrifuged at 1000 g for 20 min. The serum was then aliquoted and stored at −80 °C until further analysis.

### Metabolomics Analysis

For the untargeted metabolomics analysis, plasma metabolites were extracted from samples collected from patients with acute ischemic stroke as well as from healthy control subjects. A total of 100 µL of plasma was taken and placed into a 1.5 mL centrifuge tube along with 400 µL of an extraction solution composed of acetonitrile and methanol in a 1:1 (v/v) ratio, containing 0.02 mg mL^−1^ of the internal standard L‐2‐chlorophenylalanine. The mixture was vortexed for 30 s and subjected to low‐temperature sonication at 5 °C and 40 kHz for 30 min. To precipitate proteins, the samples were stored at −20 °C for 30 min before centrifugation at 13 000 g for 15 min at 4 °C. The supernatant was carefully collected and evaporated to dryness under a nitrogen stream. The dried residue was then reconstituted in 100 µL of a solution composed of acetonitrile and water in a 1:1 ratio, followed by a brief low‐temperature sonication for 5 min. Centrifugation was performed at 13 000 g and 4 °C for another 10 min to collect the supernatant, which was transferred to sample vials for subsequent LC‐MS/MS analysis.

The LC‐MS/MS analysis was executed using a Thermo UHPLC‐Exploris240 system equipped with an ACQUITY HSS T3 column (100 mm × 2.1 mm i.d., 1.8 µm; Waters, USA) at Majorbio Bio‐Pharm Technology Co. Ltd. (Shanghai, China). The mobile phase consisted of solvent A (0.1% formic acid in water) and solvent B (0.1% formic acid in a mixture of acetonitrile and isopropanol). The gradient for positive ion mode separation was as follows: 0–3 min: B increased from 0% to 20%; 3–4.5 min: B increased from 20% to 35%; 4.5–5 min: B increased from 35% to 100%; 5–6.3 min: B held at 100%; 6.3–6.4 min: B decreased back to 0%; and 6.4–8 min: B maintained at 0%. In negative ion mode, the gradient was similar, with slight variations in percentages. The flow rate was fixed at 0.40 mL min^−1^ with the column temperature maintained at 40 °C.

Mass spectrometric data were collected using a Thermo UHPLC‐Exploris240 Mass Spectrometer equipped with an electrospray ionization (ESI) source. Conditions for ionization included a heated auxiliary gas temperature of 350 °C, a capillary temperature of 320 °C, sheath gas flow set to 60 psi, and ion‐spray voltage settings of −3000 V for negative mode and 3400 V for positive mode. Normalized collision energy was set to run at values of 20‐40‐60 eV for MS/MS. Full MS resolution was established at 60 000 with MS/MS resolution at 15 000. The data collection employed Data Dependent Acquisition (DDA) mode, covering a mass range of 70–1050.

Preprocessing of UHPLC‐MS raw data was executed using Progenesis QI software, which involved baseline filtering, peak identification, integration, retention time correction, and alignment of peak data, resulting in a data matrix that included sample identifiers, m/z values, retention times, and peak intensities. The identification of metabolites was achieved using databases such as HMDB, Metlin, and a self‐compiled Majorbio Database (MJDB). The resultant data matrix was uploaded to the Majorbio cloud platform for further analysis, which included filtering to retain at least 80% of detectable metabolic features in each sample set. Missing values were filled using the minimum value approach, and normalization to the sum was performed to mitigate variations caused by sample handling and instrument fluctuations. Variables with a relative standard deviation (RSD) exceeding 30% in QC samples were excluded from the analysis, and the data underwent log10 transformation to prepare the final dataset. Principal Component Analysis (PCA) and Orthogonal Partial Least Squares Discriminant Analysis were conducted utilizing the R package “ropls” (Version 1.6.2), along with seven‐cycle interactive validation to assess model stability. Metabolites with a Variable Importance in Projection score greater than 1 and a P‐value less than 0.05 were classified as significantly different based on their relevance in the OPLS‐DA model. These differential metabolites were subsequently analyzed for metabolic pathways using KEGG enrichment analysis, with the Python “scipy.stats” package facilitating the exploration of relevant biological pathways associated with the experimental treatments.^[^ ^62^ ^]^

For the targeted metabolomic analysis of the tryptophan pathway, brain tissues from stroke mice were analyzed for metabolite extraction (brain tissues were collected after transcardial perfusion with saline). A total of 34 tryptophan pathway standards were accurately weighed, and the corresponding standard solutions were prepared in methanol. A mixed standard solution of varying concentrations was created, and 25 mg of brain tissue was sampled in a tube. To this, 10 µL of an internal standard solution (Trp‐D5, 4000 ng mL^−1^) and 190 µL of extraction solvent (methanol/water in a 4:1 ratio) were added. The samples were then homogenized using a high‐throughput tissue crusher at −10 °C for 6 min, followed by sonication for 30 min at 5 °C. Post‐sonication, samples were stored at −20 °C for protein precipitation. Following centrifugation at 13 000 g at 4 °C for 15 min, the supernatant was injected into the LC‐MS/MS system for analysis.

This analysis was performed on a Nexera Series LC‐40 system coupled with a QTRAP 6500+ mass spectrometer (Sciex, USA) at Majorbio Bio‐Pharm Technology Co. Ltd. The separation of metabolites was achieved using an ACQUITY UPLC HSS T3 (2.1 × 150 mm, 1.8 µm) column at 40 °C with a flow rate of 1 mL min^−1^. The elution gradient over a total duration of 18 min involved a mixture of water containing 0.1% formic acid (solvent A) and 100% acetonitrile in a similar acidified water solution (solvent B). The source temperature for mass spectrometric detection was set at 550 °C, with additional settings for both curtain and ion source gases calibrated appropriately. Quantitation of the metabolite concentrations was performed utilizing a linear regression standard curve based on the data collected during LC‐MS analysis.

### Animals and Models of Stroke

Male C57BL/6 mice (age: 8–10 weeks, weight: 20–24 g) were procured from SPF (Beijing) Biotechnology Co. Ltd. The mice were maintained under controlled conditions, on a 12 h light/dark cycle, at 55% ± 5% humidity and a temperature of 22 °C ± 2 °C, with ad libitum access to food and water. Prior to experimental procedures, animals were acclimatized to their environment for a minimum of three days. All protocols adhered to the NIH standards for the care and use of laboratory animals and followed the ARRIVE guidelines for reporting experiments.^[^ ^63^, ^64^ ^]^ The study received approval from the Animal Welfare Ethics Committee of Beijing Institute of Neurosurgery (Approval No. BNI202405004, Beijing, China).

To induce focal cerebral cortical ischemia, methods involved the permanent occlusion of both the right distal middle cerebral artery (MCA) and the common carotid artery (CCA), adhering to the procedural guidelines for distal middle cerebral artery occlusion previously established in the literature.^[^ ^8^ ^]^ An intraperitoneal injection of Avertin (400 mg kg^−1^, Sigma–Aldrich, Cat# T48402‐25G) was used for anesthesia. An incision was made in the neck to expose the right common carotid artery, which was subsequently ligated with an 8‐0 silk surgical suture. A small hole was then drilled in the skull to access the distal MCA, which was coagulated using a cauterizer under a microscope. Throughout the procedure, the animals' body temperature was diligently maintained at 37.5 °C ± 0.5 °C. Control sham‐operated mice underwent similar exposure of the CCA, but the artery was not occluded, nor was the distal MCA coagulated.

### 3‐HKA Administration

The 3‐HKA dihydrochloride was synthesized by TargetMol, USA (purity≥98%, #T68182L). The mice were randomly divided using a random number table generated by SPSS software version 27.0. Following the induction of MCAO, mice received intraperitoneal injections of 3‐HKA at doses of 20, 40, and 80 mg kg^−1^ (diluted in 0.9% saline). These administrations commenced immediately post‐surgery and were continued once daily for a duration of 14 days. Intraperitoneal injection adhered to prescribed protocols and the aseptic principle in order to prevent complications like peritonitis. Mice were divided into 3 groups following the principle of randomization (random number): Sham group: sham‐operated mice; MCAO+Vehicle group: mice were subjected to MCAO and vehicle (0.9% saline) treatment; MCAO+3‐HKA group: mice were subjected to MCAO and 3‐HKA treatment.

### Neurobehavioral Tests

Neurobehavioral assessments were conducted by an investigator who remained blinded to the treatment groups. Evaluations occurred prior to MCAO and continued up to 28 days post‐MCAO. Sensorimotor impairments were assessed using the rota‐rod test, mNSS, adhesive removal test, and gait analysis.

Rota‐rod Test. The rota‐rod apparatus was utilized to evaluate the motor learning and coordination abilities of mice. Prior to MCAO, subjects underwent a 5‐day training regimen. Each mouse was placed on a fixed‐speed (4 rpm) rotating rod three times daily, with 15 min intervals between trials. Only those mice that remained on the rod for 60 s or longer were included in the study. The assessment involved placing the mice on a rod that accelerated from 4 to 40 rpm over 4 min, repeated for three trials. The final score represented the average duration each mouse successfully maintained balance on the rod across all trials.

The mNSS offers a comprehensive evaluation of motor, sensory, reflexive, and balance functions, rated on a scale of 0 to 18 (where 0 indicates normal performance and 18 corresponds to maximum deficit). Subjects received one point for each reflex not exhibited or each task they could not perform, with higher scores indicating greater neurological impairment.

Adhesive Removal Test. In this assessment, a 3 mm x 3 mm piece of adhesive tape was affixed to the palmar area of the contralateral forepaw. The time taken for the mouse to contact and subsequently remove the adhesive was recorded. Each subject underwent three trials per day from three days before MCAO until designated postoperative time points. The average performance from the three trials the day before MCAO established the baseline.

Gait Analysis. The gait assessment was conducted using the CatWalk XT system (version 10.6, Noldus Information Technology), designed to evaluate motor performance and footfall patterns in ischemic mice, following established protocols.^[^ ^65^, ^66^ ^]^ Each subject was allowed to traverse a corridor featuring a glass runway, evenly illuminated from below to enhance visibility. Movement capture was facilitated by a camera operating at a frame rate of 100 frames per second for a duration of 20 s, enabling comprehensive tracking of all four limbs. Subsequent analysis was performed utilizing CatWalk XT software. A successful walking trial was defined by the ability of the mice to maintain a steady pace without exhibiting behaviors such as stopping, grooming, or rearing. During the test, the mice were positioned on the glass pathway either at a constant speed of 8 cm ^−1^s or at their maximum achievable speed while still exhibiting optimal coordination in locomotion. Background images were systematically recorded prior to each testing session. Each mouse underwent three individual trials, with intervals exceeding 15 min between trials. Prior to MCAO, subjects from each experimental group participated in a 3‐day acclimatization process, during which baseline gait data were acquired via the software. Detailed parameters for the gait analysis are outlined in (**Table** **1**).

**Table 1:** Description of various parameters analyzed in this study.

Parameters	Description
Static Parameters	
Print Area	The total surface area of the complete paw print.
Dynamic Parameters	
Average Run Speed	Calculated by dividing the distance covered by the animal's center by the time taken to cover that distance.
Maximum Lateral Deviation	The farthest distance during the stance that the foot attained relative to the long body axis (nose to tail axis). This is the essentially the farthest the foot got away from the body axis.
Maximum Longitudinal Deviation	The farthest distance during the stance that the foot attained relative to the short body axis (waist axis). This is the essentially the farthest the foot got away from the waist.
Stride Length	Stride Length is the distance between successive placements of the same paw.
Combined Paw Parameters	
Base of Support Front Paws	The mean distance measured between the front paws during locomotion.
Base of Support Hind Paws	The mean distance measured between the hind paws during locomotion.
Base of Support Left Paws	The mean distance measured between the left front and left rear paws.

LF: left front; LR: left rear; RF: right front.

### Brain Infarct Volume

To assess the infarct volume at 7 days post‐MCAO, 2,3,5‐Triphenyltetrazolium chloride (TTC) staining was utilized. Brain tissue was sectioned into 8 coronal slices, each measuring 1 mm in thickness. The slices were incubated in a 2% TTC solution at 37 °C for 20 min, followed by fixation in 4% paraformaldehyde (PFA). Normal brain tissue exhibited red staining, while infarcted areas appeared pale. The infarct volume was quantified using ImageJ software.

(1) $$\begin{array}{c} \begin{array}{c} Percentage\;hemisphereisphere\;lesion\;volume\;after\;correction\;of\;edema=\\ \frac{total\;infarct\;volume\;-\;\left(ipsilateral\;hemisphere\;volume\;-\;contralateral\;hemisphere\;volume\right)}{contralateral\;hemisphere\;volume}*100\% \end{array} \end{array}$$

### Cerebral Blood Flow Measurement

The local CBF was quantified utilizing a 2D laser speckle imager (PeriCam PSI, Perimed, Stockholm, Sweden) as described.^[^ ^65^ ^]^ Initially, the mice were anesthetized and fixed in a stereotaxic head frame. A midline incision was made in the scalp to allow for exposure of the skull. The laser speckle camera was positioned ≈10 cm above the exposed skull surface. 2D microcirculation images were acquired at multiple time points: 15 min prior to MCAO, immediately following MCAO, and at intervals of 3, 7, 14, and 28 days post‐MCAO. For each subject, five sequential images were recorded at every designated time point. Symmetrical elliptical regions of interest were delineated in both the ischemic and contralateral non‐ischemic hemispheres for the purpose of image data analysis. The analysis of dynamic CBF fluctuations was performed using PIMSoft software. The CBF ratio was initially calculated as the ischemic CBF in relation to the non‐ischemic CBF, and subsequently, this ratio was normalized against the pre‐MCAO baseline, yielding relative CBF values for each individual animal.

### Immunofluorescence Staining

Mice were anesthetized and subsequently subjected to transcardial perfusion with saline, followed by cold 4% PFA in 0.1 M phosphate‐buffered saline (PBS). Upon collection, the brains were cryoprotected in 30% sucrose in PBS for a duration of two days. Serial coronal sections were then obtained at a thickness of 15 µm using a cryotome (Thermo Scientific, USA). The acquired sections underwent a permeabilization step with 0.3% Triton X‐100 for 15 min and were then blocked with 10% normal donkey serum at 37 °C for 1 h. Following this, primary antibodies were applied and incubated overnight at 4 °C. The primary antibodies utilized for the immunofluorescence included rat anti‐CD31 (1:50, BD Biosciences #550274), mouse anti‐GFAP (1:200, Boster #BM0055), rabbit anti‐S100a10 (1:300, Invitrogen #PA5‐95505), mouse anti‐NeuN (1:200, Abcam #ab104224), goat anti‐Iba1 (1:200, Abcam #ab289874), and rabbit anti‐AIM2 (1:200, Invitrogen #PA5‐91996). On the second day, the sections were washed with PBS and subsequently incubated with the appropriate secondary antibodies (1:500 or 1:800, Alexa Fluor 488 or 594, Jackson ImmunoResearch) at 37 °C for 1 h. Negative control sections were treated identically, omitting the primary antibodies. Images of the labeled sections were captured using laser scanning confocal microscopy with either a 20 × /0.7NA or 40 × /1.0NA objective (Zeiss, German). For each region of interest, images 3–4 sections per mouse were analyzed. The vascular density (%), vascular length (mm), branching index (junctions/mm^2^) and lacunarity were quantified with the Angio Tool (National Cancer Institute, USA) in a blinded manner^[^ ^67^ ^]^. GFAP‐positive vessels, S100a10^+^/GFAP^+^ cell counts, NeuN^+^/AIM2^+^ cells, GFAP^+^/AIM2^+^ cells, Iba1^+^/AIM2^+^ cells and CD31^+^/AIM2^+^ cells were analyzed using ImageJ software.

To examine the proliferation of new cells, the S‐phase marker 5‐bromo‐2′‐deoxyuridine (BrdU, Sigma–Aldrich Cat# T48402) was employed. Mice received intraperitoneal injections of BrdU at a dosage of 50 mg kg d^−1^, starting 24 h post‐MCAO and continuing for 14 days. Mice were euthanized 14 days after MCAO, and coronal sections of the brain were prepared. For BrdU detection, the sections were subjected to denaturation using 2N hydrochloric acid (HCl) at 37 °C for 30 min, followed by neutralization in boric acid (pH 8.5) for three 5 min intervals at room temperature. Following this, sections were blocked with 10% normal donkey serum for 1 h at 37 °C. Subsequently, slices were incubated with sheep anti‐BrdU (1:200, Abcam #ab1893) and rabbit anti‐CD31 (1:100, Abcam #ab28364). On the second day, slices were washed with PBS, and appropriate secondary antibodies (Alexa Fluor 488, Alexa Fluor 594, or DyLight 405; Jackson ImmunoResearch, USA) were applied for 1 h at 37 °C. Images from 3–4 sections per mouse were analyzed for specific regions of interest, with BrdU+/CD31+ cell counts determined using ImageJ software. 3D images were processed using Imaris software (Bitplane).

### In‐Vivo Two‐Photon Microscopy

Mice were anesthetized and maintained on a heating plate at 37 ± 0.5 °C. Following fixation in a custom‐made head holder, a craniotomy was performed over the right forelimb area of the motor cortex, previously defined by intracortical mapping experiments, using a high‐speed micro drill.^[^ ^68^ ^]^ The exposed skull was replaced with a 3 mm diameter sterile coverslip (Warner Instruments, #64‐0720) and secured with cyanoacrylate (3 M Vetbond Tissue Adhesive, #1469) and dental cement. The cranial windows were positioned ≈2 mm rostral to bregma and 0.5 mm lateral to the midline. Imaging was conducted through these cranial windows using a two‐photon laser‐scanning microscope (Zeiss LSM880, Germany) equipped with a × 20 Zeiss W Plan objective. Two‐photon excitation was achieved using a Ti:sapphire laser (MaiTai DeepSee, Spectra Physics, USA). Microvascular perfusion was assessed following an intravenous injection of 0.1 mL of 10 mg mL^−1^ FITC‐dextran (2000 kDa, Sigma–Aldrich; St. Louis, MO). Vascular images were captured from 200 to 400 µm below the cortical surface (numerical aperture 2.0; zoom magnification 1 ×; time series mode; speed 7; image size 425.1 µm × 425.1 µm; resolution 512 × 512; excitation wavelength 800 nm). Images were reconstructed using ZEN 2 blue edition (Zeiss, Germany) software. Blood vessel segmentation and network analysis were performed with the ImageJ plugin TubeAnalyst v5.^[^ ^2^ ^]^

Cerebrovascular permeability of FITC‐dextran (40 kDa) was assessed at 14 days post‐cerebral ischemia. Time‐lapse imaging was performed at 10, 20, and 30 min following an intravenous injection of 0.1 mL of 10 mg mL^−1^ FITC‐dextran (40 kDa, Sigma–Aldrich; St. Louis, MO). Automatic vessel segmentation was carried out on each individual Z‐slice at the initial time point (when the blood‐brain barrier was impermeable to the dextran) to distinguish intravascular and extravascular compartments. Intravascular ROIs were masked based on segmented vessels, and extravascular ROIs were identified by subtracting intravascular ROIs from the imaging field of view. Fluorescent intensity for each compartment, Ii(t) and Ie(t), was calculated by averaging pixel intensity within the compartment ROIs over the entire depth.

To measure permeability based on changes in fluorescent intensity within the intravascular and extravascular spaces, the following formula was applied:

(2) $$\alpha \left(t\right)=\frac{dIe/dt}{\frac{I_{i}\left(t\right)}{1-HCT}-\frac{Ie\left(t\right)}{v_{e/v_{i}}}}$$

where Ve/Vi represents the volume fraction between extravascular and intravascular compartments, obtained from vessel segmentation. A hematocrit (HCT) level of 45% was assigned to account for the average hematocrit level of all blood vessels within the imaging field of view.^[^ ^34^, ^69^ ^]^ This method provides a precise and quantitative approach to evaluate cerebrovascular permeability post‐ischemia, leveraging advanced imaging and analytical techniques to obtain reliable data.

### Evans Blue Extravasation

To assess Evans blue extravasation, a 2% Evans blue solution in saline (4 mL kg^−1^, Sigma) was administered via tail vein injection 24 h before the mice were anesthetized. Following anesthesia, both the ipsilateral and contralateral hemispheres were excised for analysis. Each hemisphere was homogenized in 1 mL of trichloroacetic acid and subjected to centrifugation at 21 000 × g for 20 min. The absorbance of the resulting supernatant was measured at 620 nm using a spectrophotometer (TECAN, Austria) to quantify the Evans blue concentration.

### Quantitative Real‐Time PCR (qRT‐PCR)

Total RNA was extracted from brain tissues and astrocyte cells using the Trizol reagent (Invitrogen, CA) in accordance with the manufacturer's protocol. For cDNA synthesis, we utilized the PrimeScript First Strand cDNA Synthesis Kit (Takara, Japan). Quantitative real‐time PCR analysis was carried out utilizing the PowerUp SYBR Green Master Mix (Thermo Fisher Scientific, USA) in conjunction with the LightCycler 480 PCR System (Roche, USA). We employed the NCBI Primer‐BLAST tool (http://www.ncbi.nlm.nih.gov/tools/primer‐blast/) for the design of primers, ensuring the selection of pairs with minimal likelihood of non‐specific amplification, as predicted by the software. The efficiency of all primers was maintained within a range of 90%–105%. Gene expression levels were normalized to *Gapdh* and calculated using the 2^‐ΔΔCt^ method. Primer sequences for these genes are provided in **Table** **2**.

**Table 2:** Mouse primer sequences.

Gene	Forward primer (5′‐ 3′)	Reverse primer (5′‐ 3′)
*Aim2*	CTGTCTGCCGCCATGCTTCC	ACTGTCTTGTTCCCACTGCCTTTG
*Nlrp3*	GGACAGCCTTGAAGAAGAGTGGATG	TGCTTGCTTGGATGCTCCTTGAC
*Nlrp1a*	GCCTCACATCCACATACTGCTCAC	CTGACTGCTGTCTCTGCTGCTTC
*Nlrc4*	GCGAGTCTGGCAAAGGGAAGTC	CCGTGGTGGTGGTGACAATGAC
*Gapdh*	TTGATGGCAACAATCTCCAC	CGTCCCGTAGACAAAATGGT
*H2‐T23*	GGACCGCGAATGACATAGC	GCACCTCAGGGTGACTTCAT
*Serping1*	ACAGCCCCCTCTGAATTCTT	GGATGCTCTCCAAGTTGCTC
*H2‐D1*	TCCGAGATTGTAAAGCGTGAAGA	ACAGGGCAGTGCAGGGATAG
*Ggta1*	GTGAACAGCATGAGGGGTTT	GTTTTGTTGCCTCTGGGTGT
*Iigp1*	GGGGCAATAGCTCATTGGTA	ACCTCGAAGACATCCCCTTT
*Gbp2*	GGGGTCACTGTCTGACCACT	GGGAAACCTGGGATGAGATT
*Fbln5*	CTTCAGATGCAAGCAACAA	AGGCAGTGTCAGAGGCCTTA
*Ugt1a*	CCTATGGGTCACTTGCCACT	AAAACCATGTTGGGCATGAT
*Fkbp5*	TCGTTCCTCCTCGCAGCCTTC	CGTTGTGCTCCTTCGCCTTCC
*Psmb8*	CAGTCCTGAAGAGGCCTACG	CACTTTCACCCAACCGTCTT
*C3*	CACACCGAAGAAGACTGCCTGAC	CTGACTTGATGACCTGCTGGATGG
*Amigo2*	GAGGCGACCATAATGTCGTT	GCATCCAACAGTCCGATTCT
*Clcf1*	CTTCAATCCTCCTCGACTGG	TACGTCGGAGTTCAGCTGTG
*Tgm1*	CTGTTGGTCCCGTCCCAAA	GGACCTTCCATTGTGCCTGG
*Ptx3*	AGTGGCTGAGACCTCGGATGAC	AGACACTGTCGCCTCGGATCG
*S100a10*	CGAGATGGCAAAGTGGGCTTCC	TTCCTAAGGGTCCTGATCTGCTCAC
*Sphk1*	GATGCATGAGGTGGTGAATG	TGCTCGTACCCAGCATAGTG
*Cd109*	CACAGTCGGGAGCCCTAAAG	GCAGCGATTTCGATGTCCAC
*Ptgs2*	CTGGTGCCTGGTCTGATGATGTATG	GGGTGCCAGTGATAGAGTGTGTTG
*Emp1*	GAGACACTGGCCAGAAAAGC	TAAAAGGCAAGGGAATGCAC
*Slc10a6*	CATTCTCAAGGTCGGAGCCATTCTG	GCCTCCTCTTGTATGCCTGATATGC
*Tm4sf1*	GCCCAAGCATATTGTGGAGT	AGGGTAGGATGTGGCACAAG
*B3gnt5*	CGTGGGGCAATGAGAACTAT	CCCAGCTGAACTGAAGAAGG
*Cd14*	GGACTGATCTCAGCCCTCTG	GCTTCAGCCCAGTGAAAGAC

### Proteomics Analysis

The brain tissues were suspended in protein lysis buffer (8 M urea, 1% SDS) containing appropriate protease inhibitors to inhibit protease activity. The mixture was then processed using a high‐flux tissue grinder for 3 cycles of 40 s each. Subsequently, the mixture was incubated on ice for 30 min, with vortexing for 5–10 s every 5 min. The samples were centrifuged at 16 000 g for 30 min at 4 °C. Protein concentration in the collected supernatant was measured using the Bicinchoninic Acid (BCA) method with the BCA Protein Assay Kit (Thermo Scientific), following the kit protocol. 100 µg of protein was re‐suspended in Triethylammonium bicarbonate buffer (TEAB) to a final concentration of 100 mM. The mixture was reduced with Tris (2‐carboxyethyl) phosphine (TCEP) to a final concentration of 10 mM at 37 °C for 60 min, and alkylated with iodoacetamide (IAM) to a final concentration of 40 mM at room temperature for 40 min in the dark. After centrifugation at 10 000 g for 20 min at 4 °C, the pellet was re‐suspended in 100 µL TEAB buffer (100 mM). Trypsin was added at a 1:50 trypsin‐to‐protein mass ratio and incubated at 37 °C overnight. Post‐trypsin digestion, peptides were vacuum‐dried. They were then re‐solubilized in 0.1% trifluoroacetic acid (TFA), desalted using HLB columns, and vacuum‐dried again. The peptide concentration was determined using the Thermo Fisher Scientific Peptide Quantification Kit (item #23 275). Equivalent amounts of peptides were re‐dissolved in spectrometry loading buffer (2% acetonitrile with 0.1% formic acid) containing appropriate iRT peptides for retention time calibration. The peptides were analyzed using an EASY‐nLC 1200 system coupled with a Q Exactive HF‐X quadrupole orbitrap mass spectrometer (Thermo, USA) at Majorbio Bio‐Pharm Technology Co. Ltd. (Shanghai, China). The C18‐reversed phase column (75 µm x 25 cm, Thermo, USA) was equilibrated with solvent A (2% ACN with 0.1% formic acid) and solvent B (80% ACN with 0.1% formic acid) at a flow rate of 300 nL min^−1^. Peptides were eluted with the following gradient: 0–70 min, 5%–23% B; 70–90 min, 23%‐29% B; 90–100 min, 29%–38% B; 100–102 min, 38%–48% B; 102–103 min, 48%–100% B; 103–120 min, maintain 100% B. The Q Exactive HF‐X operated in data‐independent acquisition (DIA) mode, switching automatically between full scan MS and MS/MS acquisition. Full scan MS spectra (m/z 300–1500) were acquired, followed by fragmentation of all precursor ions in the collision cell by HCD. DIA was performed with a variable isolation window, each overlapping by 1 m z^−1^, with a total of 40 windows. DIA raw data were imported into Spectronaut software (Version 14) for library search analysis. Retention times were corrected using iRT peptides, and quantitative analysis was performed by selecting 6 peptides per protein and 3 fragment ions per peptide. The parameters included: Protein FDR ≤ 0.01, Peptide FDR ≤ 0.01, Peptide Confidence ≥99%, XIC width ≤75 ppm. Shared peptides and modified peptides were excluded, and peak areas were calculated and summed for quantitative results. Proteomic data were analyzed using the Majorbio Cloud platform (https://cloud.majorbio.com). *P*‐values and fold change for proteins between groups were calculated using the R package “t‐test”. DEPs were identified using thresholds of fold change ratio > 1.5 and *P*‐value < 0.05. Functional annotation of identified proteins was performed using GO (http://geneontology.org/) and KEGG pathway (http://www.genome.jp/kegg/). DEPs were further subjected to GO and KEGG enrichment analysis. Protein‐protein interaction analysis was conducted using String v11.5.

### Western Blot

Proteins from brain tissue and cultured cells were extracted using Radio Immunoprecipitation Assay (RIPA) lysis buffer (Solarbio #R0020) supplemented with 1% protease inhibitor cocktail (Sigma #P8340) and 1% phosphatase inhibitor (Applygen #P1260) at 4 °C. Protein concentrations were determined using the BCA protein assay kit (Thermo Scientific, USA). Subsequently, 30 µg of protein per sample was separated by 10% SDS‐PAGE and transferred onto PVDF membranes (Roche, USA). The membranes were blocked with 5% nonfat milk in TBST for 1 h at room temperature, followed by overnight incubation at 4 °C with primary antibodies. The primary antibodies used were AIM2 (1:500, Invitrogen #PA5‐91996), pro‐caspase‐1 (1:1000, AdipoGen #AG‐20B‐0042), p20 caspase‐1 (1:1000, AdipoGen #AG‐20B‐0042), ASC (1:1000, Abcam #ab307560), and VEGF‐A (1:800, Abcam #ab46154). GAPDH (1:10 000, Bioworld Technology #AP0063) was employed as a loading control. After primary antibody incubation, membranes were washed three times with TBST and then incubated for 1 h at room temperature with Goat Anti‐Rabbit IgG (H&L) Antibody DyLight 800 Conjugated secondary antibody (1:10 000, Rockland #611‐145‐122). Protein levels were detected using an Odyssey infrared imaging system (LICOR Biosciences, USA). Band intensities were analyzed with ImageJ software, and the relative expression levels of AIM2, pro‐caspase‐1, p20 caspase‐1, ASC and VEGF‐A were quantified relative to GAPDH in both in vivo and in vitro samples.

### Cytometric Bead Array

To quantify the concentrations of IL‐1β, IL‐18, IL‐6 and TNF‐α in the supernatant of astrocyte cells, the BD Cytometric Bead Array Mouse Soluble Protein Master Buffer Kit (Becton, Dickinson and Company, USA) was employed. The procedures for preparing standards, capture beads, detection reagents, and samples, along with the setup of flow cytometer protocols and data analysis, were meticulously followed as per the kit's instructions.

In brief, the standards were reconstituted and serially diluted immediately before use. The capture beads were prepared, and 50 µl of the mixed beads were added to each assay tube, followed by the addition of 50 µl of either standards or samples. The tubes were thoroughly mixed and incubated for 1 h at room temperature in the dark. Subsequently, 50 µl of PE detection reagent was added to each tube, followed by another 1 h incubation at room temperature. The samples were then washed with 1.0 ml of wash buffer and centrifuged at 200 g for 5 min. After discarding the supernatant, the beads were resuspended in 300 µl of wash buffer. The samples were analyzed using a BD FACSAria II flow cytometer, and data were processed with FCAP Array software (version 3.0, BD Biosciences).

### Co‐Culture of Primary Astrocytes and Primary Brain Microvascular Endothelial Cells

Primary astrocytes were isolated from the forebrains of postnatal day 1–2 mice using previously validated protocols.^[^ ^22^ ^]^ Briefly, the excised forebrain cortices were enzymatically dissociated in Hibernate‐E medium (Sigma, USA) containing 2.0 mg mL^−1^ papain (Sigma, USA) at 37 °C for 10 min. The resultant cell suspension was then filtered through a 70 µm cell strainer and plated into 25 cm^2^ culture flasks pre‐coated with poly‐L‐lysine to initiate mixed‐glial cultures. Non‐adherent cells were removed after 24 h by changing the medium. The mixed‐glial cultures were maintained in low‐glucose DMEM medium (Gibco, USA) supplemented with 10% fetal bovine serum (FBS; Gibco, USA), 0.58 mg ml^−1^ L‐glutamine (Gibco, USA), and 1% penicillin‐streptomycin (Solarbio, China), with medium renewal every 1–2 days for a duration of 5–7 days. Astrocyte enrichment was achieved by orbital shaking of the cultures at 300 rpm for 12 h at 37 °C, which facilitated the detachment of non‐astrocytic glial cells. Cultures with over 95% GFAP‐positive astrocytes were selected for subsequent in vitro experiments.

For the isolation and culture of primary brain microvascular endothelial cells, cortical tissues were harvested from postnatal day 10 mice. Following the removal of the brain, meninges and large blood vessels were carefully excised by rolling the brain on sterile, dry filter paper. The brain hemispheres were then dissociated in cold D‐Hanks’ solution (Gibco, USA), and the gray matter was dissected into ≈1 mm^3^ pieces. The tissues were subjected to an initial centrifugation at 150 × g for 3 min. The tissue pellets were resuspended in a 25% bovine serum albumin (BSA) solution, which was approximately twice the volume of the precipitate. The suspension was homogenized using 25–30 pipette strokes, and then centrifuged at 600 × g for 10 min. The resulting microvessel pellet, found at the bottom of the tube, was collected and subjected to further enzymatic digestion. The digestion process involved incubating the microvessel pellet with 0.1% collagenase type II at 37 °C for 35 min with intermittent agitation. Post‐digestion, the solution was gently resuspended and centrifuged at 150 × g for 5 min. The resulting pellet, containing beaded microvessel fragments and individual endothelial cells, was collected and cultured. Typically, tissue from two brains was sufficient to seed one culture flask. The isolated BMVECs were maintained in low‐glucose DMEM medium (Gibco, USA) supplemented with 10% fetal bovine serum (FBS; Gibco, USA), 0.58 mg ml^−1^ L‐glutamine (Gibco, USA), and 1% penicillin‐streptomycin (Solarbio, China). Cells were seeded into gelatin‐coated culture flasks at a density of 1 × 10⁵ cells ml^−1^ and incubated at 37 °C in a humidified atmosphere with 5% CO2. The culture medium was refreshed every 2–3 days. Cultures with over 95% CD31‐positive endothelial cells were selected for subsequent in vitro experiments.

In this study, a transwell‐based co‐culture system was employed to investigate cellular interactions under ischemic conditions. Three distinct co‐culture models were established using transwell plates (Corning, USA) (Figure S8, Supporting Information). Model 1: BMVECs were cultured exclusively in the bottom compartment of a 6‐well transwell co‐culture plate. Model 2: Purified primary astrocytes were initially seeded in the bottom compartment of a 6‐well transwell co‐culture plate. Following 24 h of culture, BMVECs were seeded onto the upper side of the transwell insert (PET membrane, 0.4 µm pore size), which was then positioned into the well. After OGD treatment, the astrocytes from the bottom compartment were harvested for western blot and qRT‐PCR analysis. Model 3: Purified primary astrocytes were seeded directly onto the upper side of the transwell insert (PET membrane, 0.4 µm pore size) within a 96‐well plate. After a 24 h incubation period, BMVECs were seeded into the lower compartment of the 96‐well transwell co‐culture plate, which had been pre‐coated with Matrigel. Following OGD treatment, a tube formation assay was conducted to assess angiogenic activity.

### OGD Model

Combined oxygen and glucose deprivation and reoxygenation were performed as an in vitro model of ischemic stroke. Briefly, for OGD treatment, primary astrocytes or BMVECs were washed and incubated with glucose‐free DMEM (Gibco, USA) supplemented with 0.58 mg ml^−1^ L‐glutamine, and 1% penicillin‐streptomycin, then cells were placed in a hypoxic chamber including 5% CO2 and 95% N2 at 37 °C for 6 h. After that, cells were incubated with normal culture medium at 37 °C for 12 h under normoxic conditions (5% CO2/95% air).

### Cell Viability Assay

Cell viability was determined using the Cell Counting Kit‐8 (CCK‐8, Dojindo, Japan) as described previously.^[^ ^8^ ^]^ For this, cells were seeded in 96‐well plates at a concentration of 2 × 10^5^ cells ml^−1^. Following the administration of OGD treatment, 10 µl of CCK‐8 reagent was introduced to each well and the plates were incubated at 37 °C for 2 h. Subsequently, absorbance at 450 nm was measured using a TECAN microplate reader. The viability of the cells in each treatment group was calculated as a percentage relative to the control group. Each experimental condition was performed in quintuplicate, and the data presented were representative of three independent experiments.

### Tube Formation Assay

The tube formation assay was performed as previously described.^[^ ^8^ ^]^ For the tube formation assay, growth factor‐reduced Matrigel (BD #356 230) was thawed and dissolved at 4 °C overnight. Subsequently, each well of a 96‐well plate was coated with 50 µl of Matrigel, followed by a 1 h incubation at 37 °C to allow for polymerization. Endothelial cells were then seeded at a density of 2 × 10⁴ cells per 100 µl in each well. Post‐OGD, the tube formation of endothelial cells was captured at 50 × magnification. The extent of tube formation was quantified by counting the number of branches using ImageJ software, and the data were normalized to the control group. The results were representative of three independent experiments.

### Enzyme Linked Immunosorbent Assay (ELISA)

The secretion of VEGF from astrocytes was evaluated using an ELISA method. Culture medium was harvested from the astrocytic plates, and the concentration of VEGF was quantified with a mouse VEGF Quantikine ELISA Kit (R&D Systems #MMV00, USA), following the manufacturer's instructions. Absorbance readings were taken at 450 nm using a TECAN microplate reader.

### In Vitro Lentivirus Transfection

To overexpress the AIM2 gene, a lentiviral vector containing Ubi‐AIM2 (NM_001013779.2)‐3FLAG‐CBh‐gcGFP‐IRES‐puromycin (LV‐AIM2) was constructed (BrainCase, China). Primary astrocytes were transduced with either LV‐AIM2 or a control lentivirus expressing GFP (LV‐NC) at a multiplicity of infection (MOI) of 5. Following a 48 h incubation post‐transduction, the cells were exposed to OGD. The efficacy of transduction was confirmed by assessing GFP expression through immunofluorescence. Furthermore, the transfection efficiency was quantitatively evaluated using qRT‐PCR.

### Intravascular Injection of Viruses

To induce AIM2 gene overexpression in astrocytes, we used an adeno‐associated virus (AAV) vector encoding the rAAV‐GfaABC1D‐mAim2‐P2A‐EGFP (AAV‐AIM2) construct from BrainCase, China, and injected it intravascularly into the tail vein of 6‐week‐old C57Bl/6 mice. Mice were immobilized in a restraining apparatus that facilitated the exposure of their tails to a light source combined with mild heating to enhance vasodilation. The tail area was thoroughly disinfected with alcohol prior to the intravenous injection of 100 µl of the viral suspension at a dose of 5 × 10^11^ Vg mouse^−1^, which contained either the AAV‐AIM2 or a control AAV vector (rAAV‐GfaABC1D‐EGFP, AAV‐NC) alone. After the injection, the animals were returned to their home cages and observed until further experimental steps. Three weeks post‐injection, the mice underwent MCAO surgery. The success of viral transduction was confirmed through EGFP expression analysis using immunofluorescence, and transfection efficiency was quantitatively assessed by qRT‐PCR.

### Statistical Analyses

Data were expressed as mean ± SD. Statistical analyses for metabolomics and proteomics were detailed in their respective sections. For normally distributed data, parametric tests were applied: Student's t‐test (two‐tailed) for comparisons between two groups, and one‐way ANOVA followed by Least Significant Difference (LSD) test (for equal variances) or Dunnett's test (for unequal variances) for comparisons involving three or more groups. For non‐normally distributed data, the Kruskal‐Wallis test followed by Dunn's post hoc analysis was used for multiple group comparisons. Experiments with two categorical independent variables and one dependent variable were analyzed using two‐way ANOVA, followed by Bonferroni's multiple comparison tests. To control Type I error across multiple comparisons, adjustments were made using LSD, Dunnett's, Dunn's, and Bonferroni's methods. The sample size (n) for statistical analyses was indicated in the corresponding figure legends. Statistical significance was set at *P* < 0.05. All statistical analyses and graphical representations were performed using SPSS software, version 27.0 (SPSS, Chicago, IL). Curve fitting was conducted using GraphPad Prism software version 8.0.2 (GraphPad Software).

## Conflict of Interest

The authors declare no conflict of interest.

## Author Contributions

J.‐M.C. wrote the original draft, provide resources, methodology, and performed investigation, formal analysis, data curation, and conceptualization. G.S. and L.‐L.Y. wrote the original draft, and performed methodology, investigation, formal analysis, and data curation. W.S. and J.‐Y.S. performed methodology, investigation, formal analysis, and data curation. A.‐C.G. and J.‐P.W. wrote, reviewed and edited. T.‐S.T., X.‐J.Z. and Q.W. performed supervision, project administration, funding acquisition, wrote, reviewed and edited.

## Supporting information



Supporting Information

Supporting Information

## Data Availability

The data that support the findings of this study are available from the corresponding author upon reasonable request.
