# Supplementary material for: 3‐HKA Promotes Vascular Remodeling After Stroke by Modulating the Activation of A1/A2 Reactive Astrocytes
Source: Adv Sci (Weinh). 2025 Jan 24;12(11):2412667. doi: 10.1002/advs.202412667 (PMC11923925; doi:10.1002/advs.202412667)
Supplement: Supplementary file 1 — Supporting Information [file ADVS-12-2412667-s001.docx]

**Supplementary Figures**


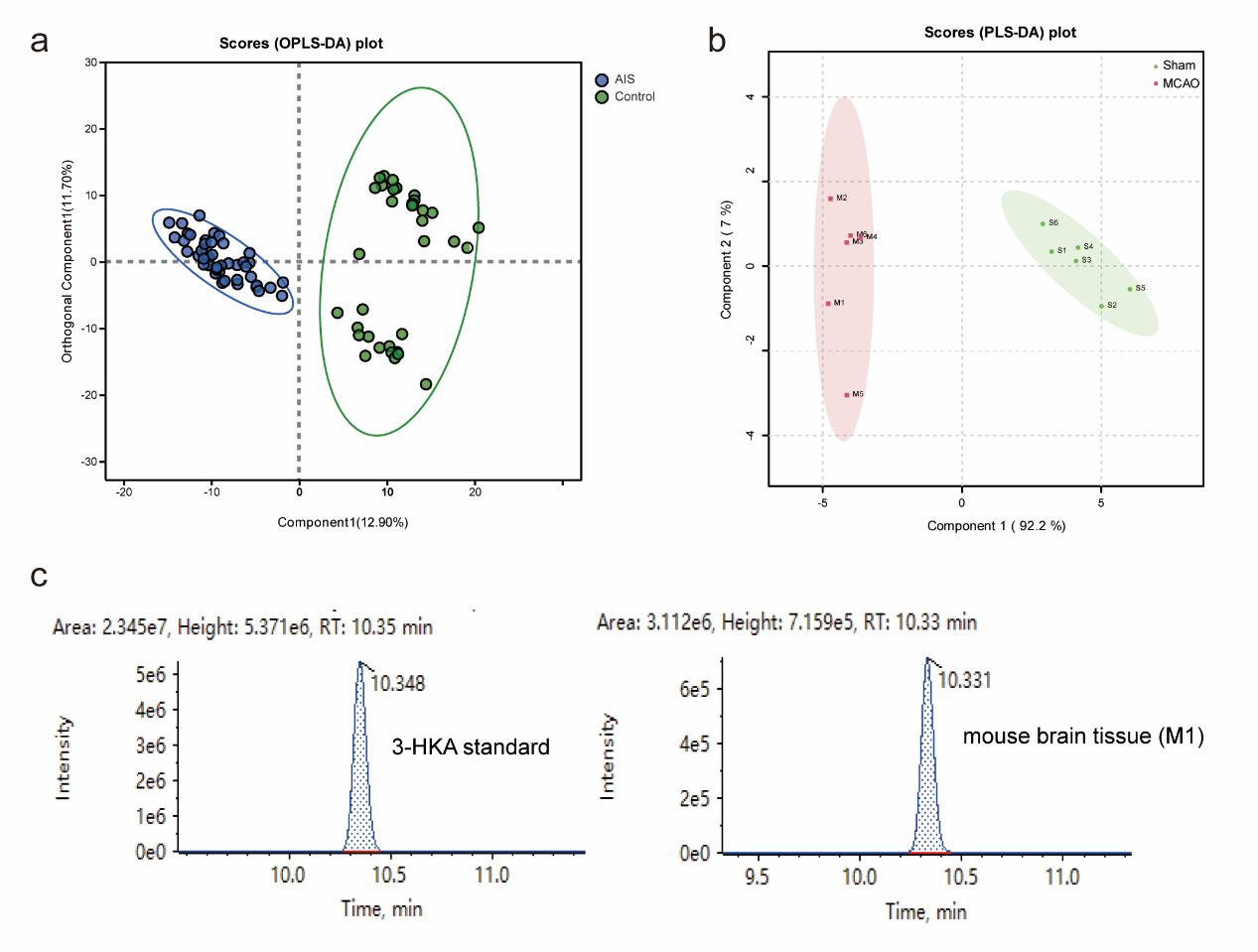


Figure S1. Identification of plasma metabolites with differential abundance associated with stroke. (a) Orthogonal partial least squares discriminant analysis (OPLS-DA) plot of patients with AIS and healthy controls. (b) Partial least squares discriminant analysis (PLS-DA) plot of mice in the MCAO and sham groups. (c) Quantify 3-HKA in the biological fluids of the 3-HKA standard and in mouse brain tissue (M1). We synthesized 3-HKA and utilized it as a standard in mass spectrometry to detect and quantify endogenous 3-HKA in biological fluids. In our extracted ion chromatogram (EIC), the retention time of the 3-HKA standard was observed at 10.348 minutes. Taking into account external environmental factors and instrument variability, the substance detected in mouse brain tissue sample (M1) with a retention time of 10.331 minutes was identified as 3-HKA.


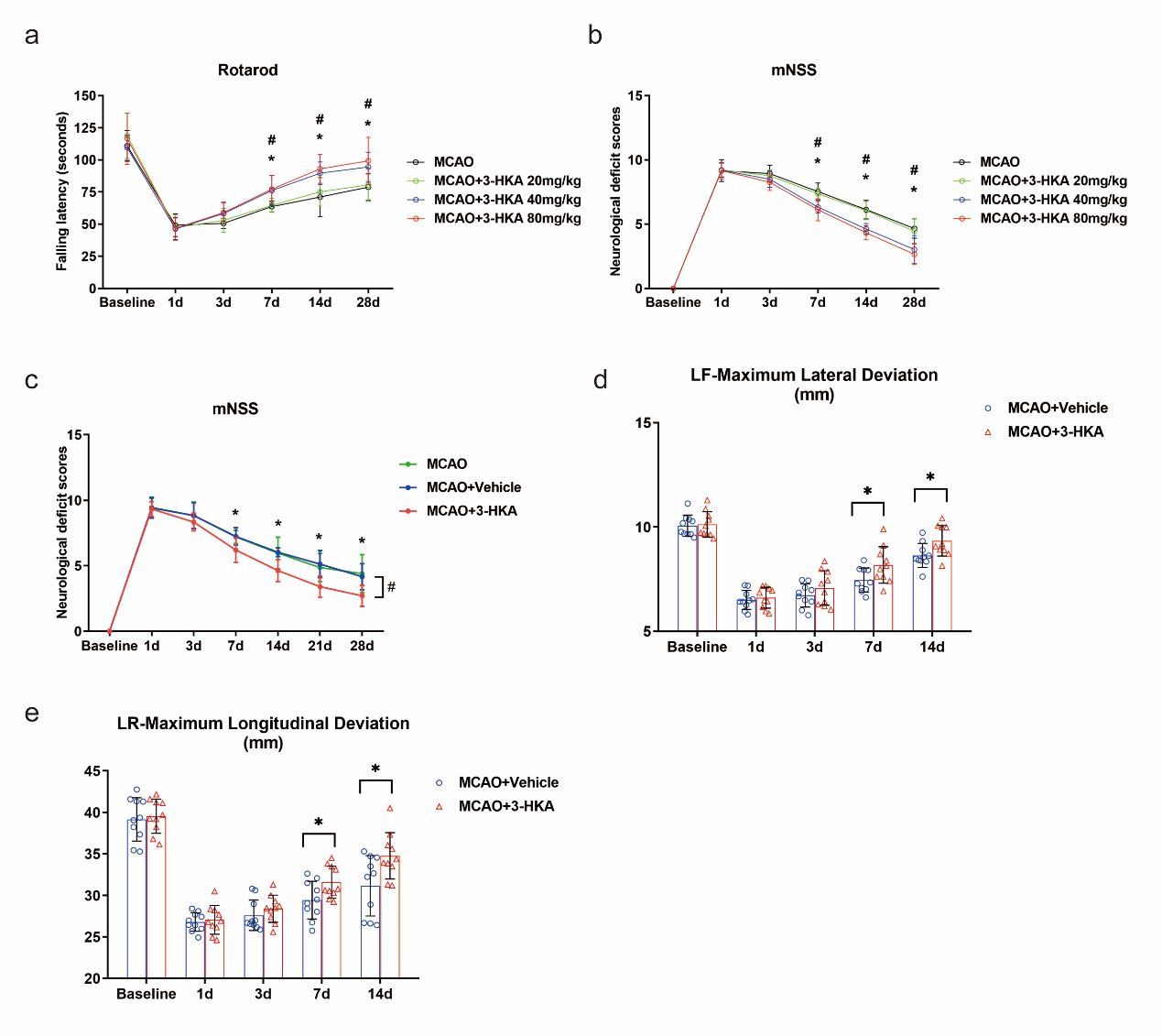


Figure S2. 3-HKA improved long-term neurological recovery. (a) Rotarod test. **P* = 0.049024 at 7 d, **P* = 0.001181 at 14 d, **P* = 0.014106 at 28 d, MCAO+3-HKA 40 mg/kg vs. MCAO. ^#^*P* = 0.015647 at 7 d, ^#^*P* = 0.000190 at 14 d, ^#^*P* = 0.001805 at 28 d, MCAO+3-HKA 80 mg/kg vs. MCAO. One-way ANOVA followed by Dunnett’s multiple comparison test was used at the 7 d time point, and LSD multiple comparison tests were used at the 14 d and 28 d time points. n = 10. (b) mNSS. **P* = 0.001408 at 7 d, **P* = 0.000004 at 14 d, **P* = 0.000268 at 28 d, MCAO+3-HKA 40 mg/kg vs. MCAO. ^#^*P* = 0.004796 at 7 d, ^#^*P* < 0.001 at 14 d, and ^#^*P* = 0.000018 at 28 d, MCAO+3-HKA 80 mg/kg vs. MCAO. One-way ANOVA followed by Dunnett’s multiple comparison test was used at 7 d, and LSD multiple comparison tests were used at 14 d and 28 d. n = 10. (c) mNSS. *P* = 0.016 at 7 d, *P* = 0.001 at 14 d, *P* = 0.001 at 21 d, and *P* = 0.00619 at 28 d; MCAO+3-HKA vs. MCAO+Vehicle by the Kruskal‒Wallis test followed by Dunn’s post hoc analysis at 7 d, 14 d, and 21 d; one‒way ANOVA followed by LSD multiple comparison test at 28 d. ^#^*P* < 0.001, MCAO+3-HKA vs. MCAO+Vehicle, by two-way ANOVA followed by Bonferroni’s multiple comparison tests (bracket). n = 10. (d) The maximum lateral deviation for the left front paw. *P* = 0.043168 at 7 d, and *P* = 0.028494 at 14 d, MCAO+3-HKA vs. MCAO+Vehicle; Student’s t test. n = 10. (e) The maximum longitudinal deviation for the left rear paw. *P* = 0.033280 at 7 d, and *P* = 0.022507 at 14 d, MCAO+3-HKA vs. MCAO+Vehicle; Student’s t test. n = 10.


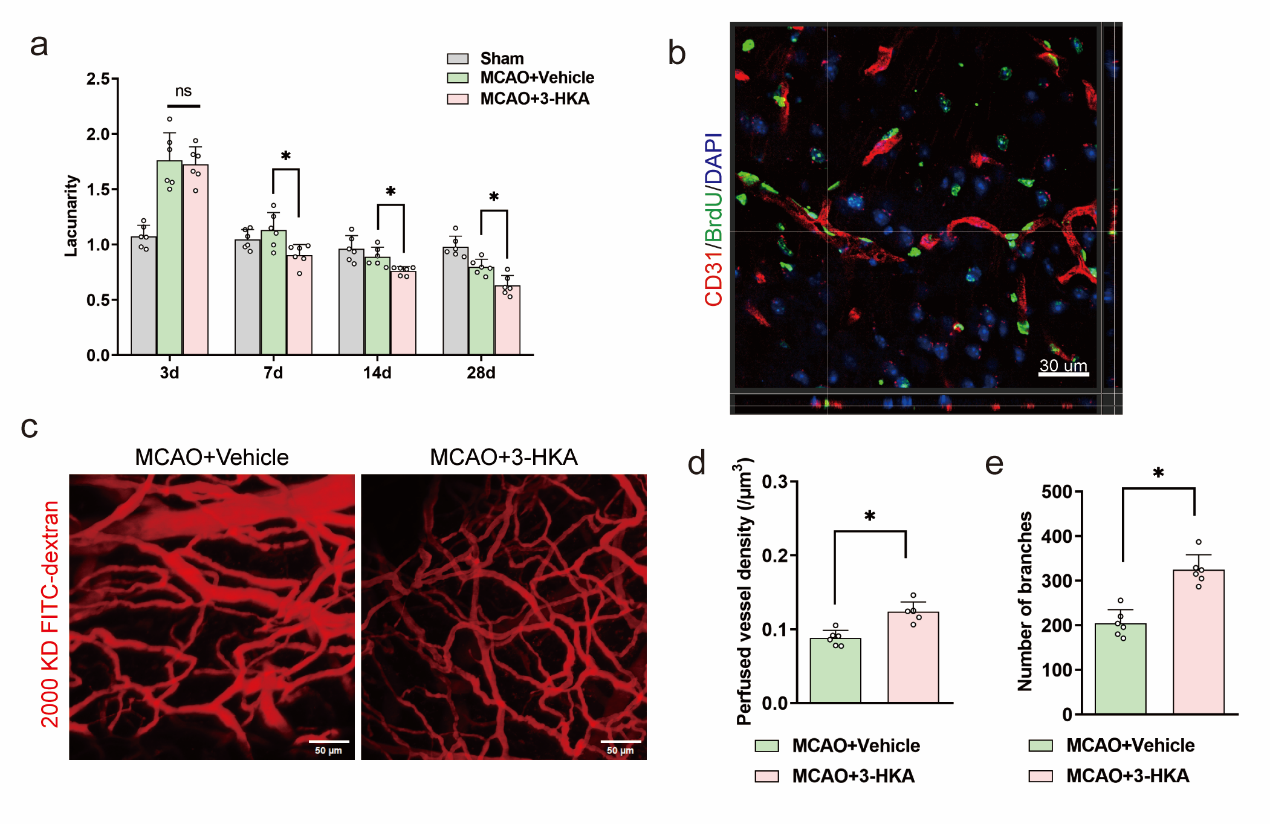


Figure S3. 3-HKA promoted vascular remodeling in ischemic mice. (a) Quantification of lacunarity. *P* = 0.004957 at 7 d, *P* = 0.023007 at 14 d, and *P* = 0.004259 at 28 d for MCAO+3-HKA vs. MCAO+Vehicle; one-way ANOVA followed by LSD multiple comparison tests (individual time points). n = 6. (b) 3D reconstruction of the orthogonal image as viewed in the x-z (bottom) and y-z (right) directions. (c-e) Representative two-photon microscopy images of perfused vessels intravenously injected with FITC-dextran (MW = 2000 kDa) in the peri-infarct region at 7 days after MCAO, with quantification of perfused vessel density (d) and the number of branches (e); Perfused vessel density, *P* = 0.000399; number of branches, *P* = 0.000077; MCAO+3-HKA vs. MCAO+Vehicle; Student’s t test. n = 6.


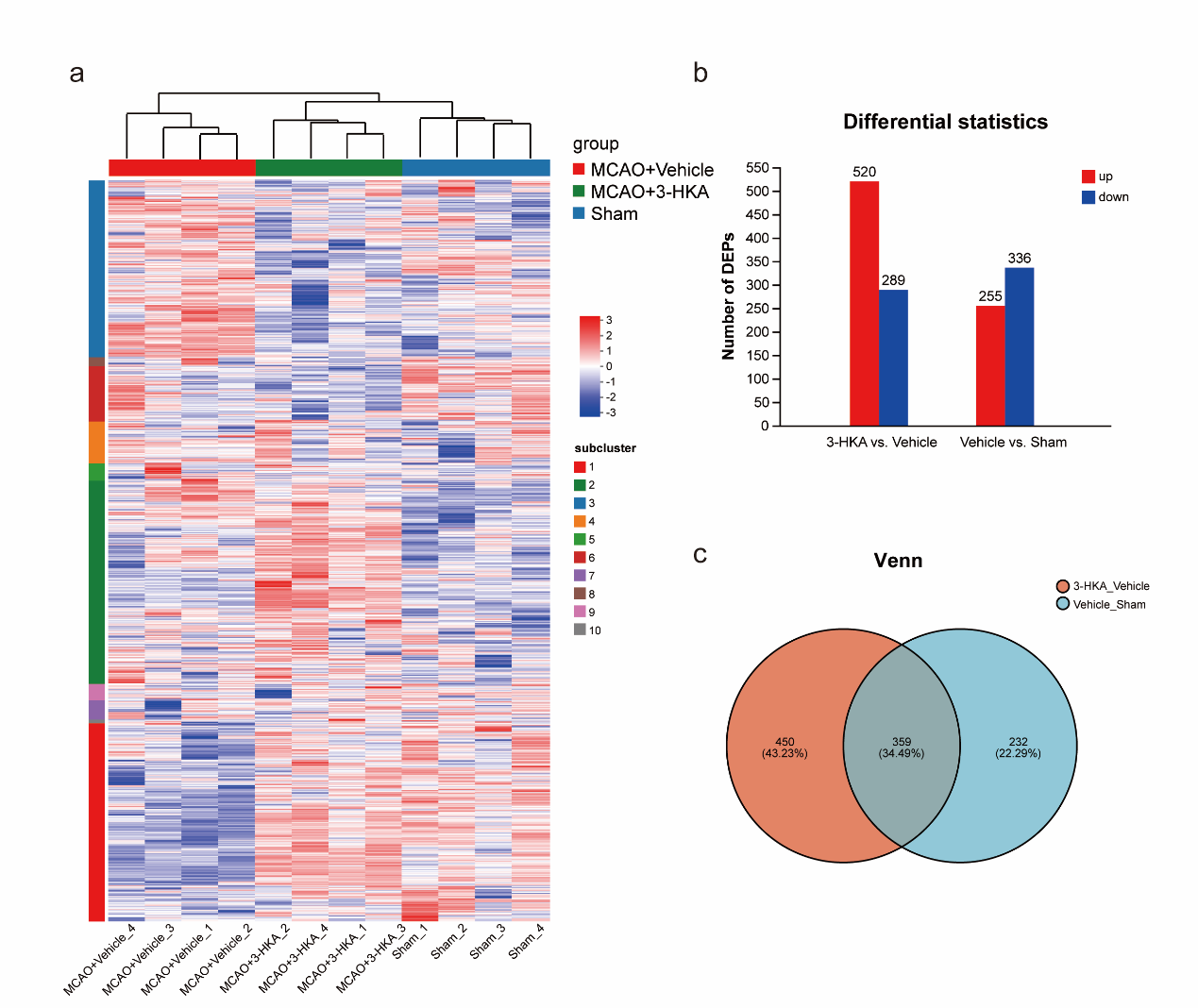


Figure S4. (a-b) Heatmap of differentially expressed proteins (DEPs) in the sham, MCAO+Vehicle, and MCAO+3-HKA groups. A total of 255 upregulated and 336 downregulated proteins were identified in the Vehicle (MCAO+Vehicle) vs. Sham comparison, and a total of 520 upregulated and 289 downregulated proteins were identified in the 3-HKA (MCAO+3-HKA) vs. Vehicle (MCAO+Vehicle) comparison. n = 4. (c) Pie chart showing the identified proteins. The number of proteins in each category is indicated; Vehicle (MCAO+Vehicle), 3-HKA (MCAO+3-HKA).


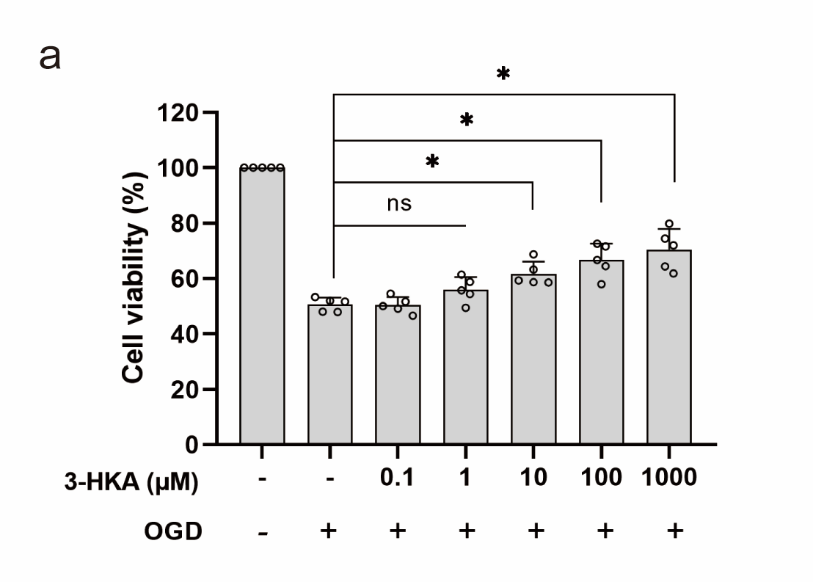


Figure S5. A cell viability assay was used to assess the minimum effective dose of 3-HKA in primary astrocytes. 3-HKA 10 μM: *P* = 0.027881 vs. OGD, 3-HKA 100 μM: *P* = 0.022942 vs. OGD, 3-HKA 1000 μM: *P* = 0.026711 vs. OGD, one-way ANOVA followed by Dunnett’s multiple comparison tests. n = 5.


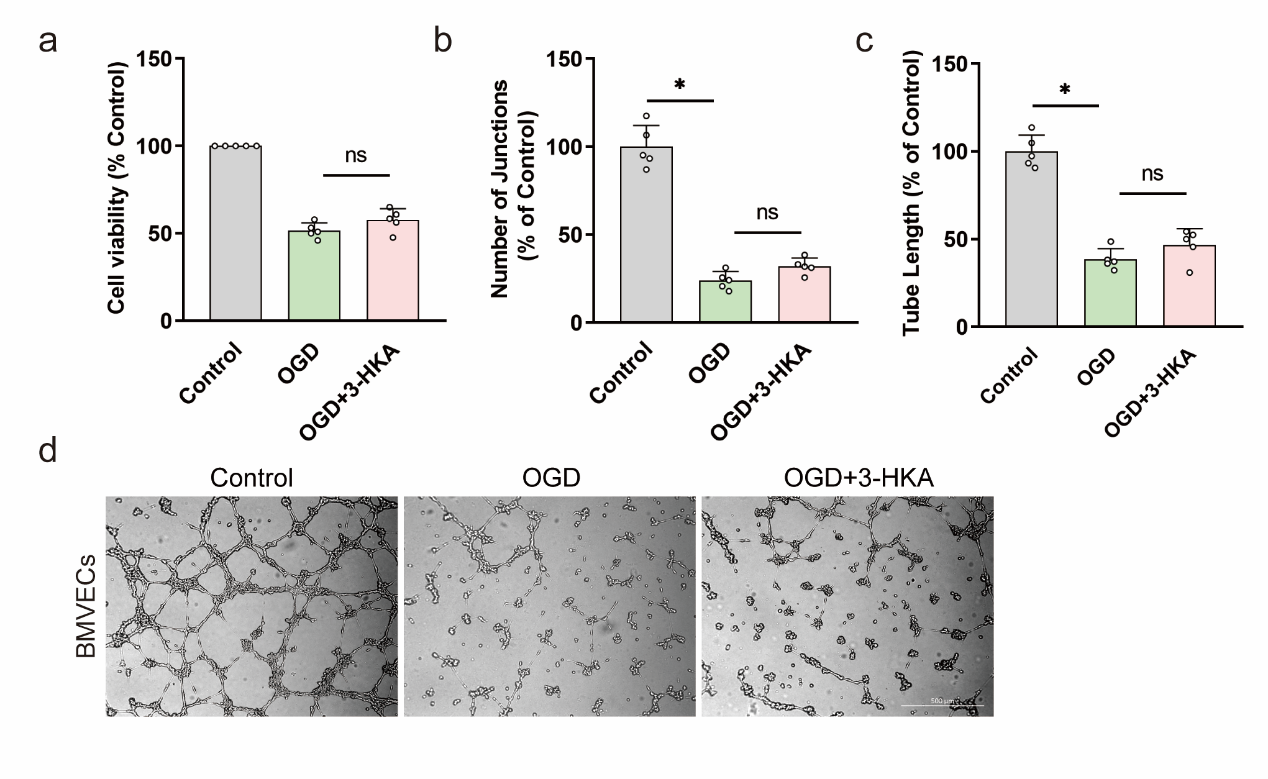


Figure S6. 3-HKA did not directly stimulate angiogenesis *in vitro*. (a) Cell viability of BMVECs. (b) Number of junctions in the capillary tube formation assay. *P* = 0.000081 (OGD vs. Control); one-way ANOVA followed by Dunnett’s multiple comparison test. n = 5. (c) Tube length in the capillary tube formation assay. *P* < 0.001 (OGD vs. Control); one-way ANOVA followed by LSD multiple comparison tests. n = 5. (d) Representative images of the capillary tube formation assay.


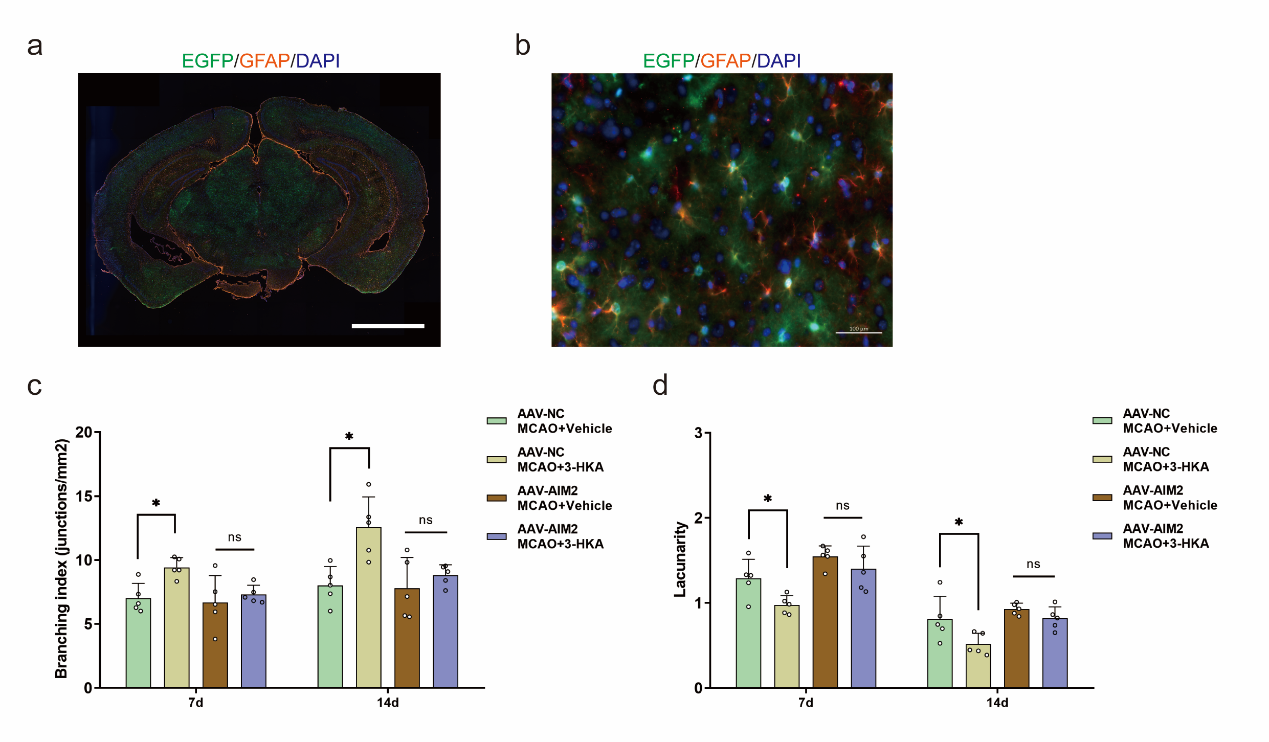


Figure S7. 3-HKA boosted post-stroke vascular remodeling *in vivo* via regulation of the A1/A2 astrocyte transition by inhibiting the activation of the AIM2 inflammasome. (a) Coronal views of fluorescent imaging in the whole brain after 3 weeks’ viral infection. EGFP (green, AAV-AIM2) and GFAP (orange, astrocyte marker). Scale bar: 2mm. (b) Representative immunostaining images of EGFP and GFAP in the cortical region of the brain slice. EGFP (green, AAV-AIM2), GFAP (orange, astrocyte marker), and DAPI (blue). Scale bar: 100μm. (c) Quantification of the vessel branching index. MCAO 7 d, *P* = 0.010705 (AAV-NC MCAO+3-HKA vs. AAV-NC MCAO+Vehicle); MCAO 14 d, *P* = 0.001541 (AAV-NC MCAO+3-HKA vs. AAV-NC MCAO+Vehicle); one-way ANOVA followed by LSD multiple comparison tests. n = 5. (d) Quantification of lacunarity. MCAO 7 d, *P* = 0.021186 (AAV-NC MCAO+3-HKA vs. AAV-NC MCAO+Vehicle); MCAO 14 d, *P* = 0.011403 (AAV-NC MCAO+3-HKA vs. AAV-NC MCAO+Vehicle); one-way ANOVA followed by LSD multiple comparison tests. n = 5.
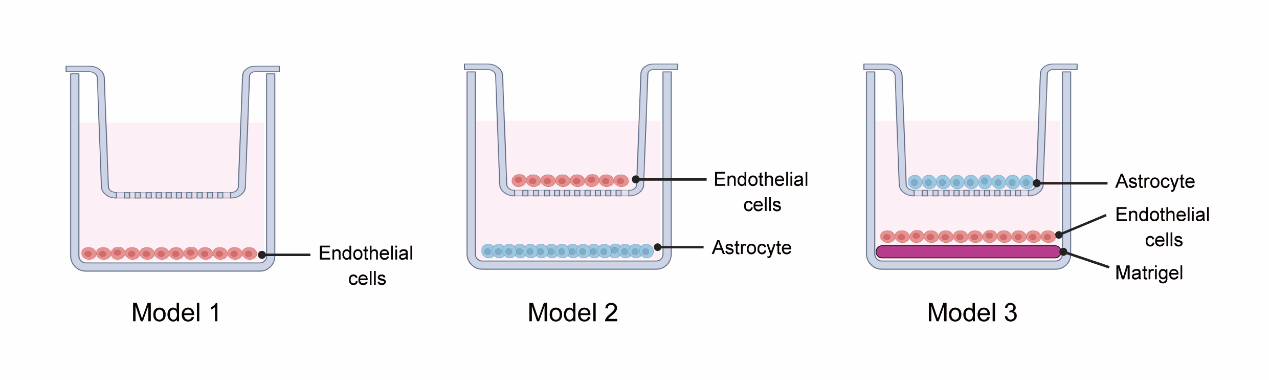
Figure S8. In this study, primary astrocytes and primary brain microvascular endothelial cells were co-cultured in three different models.
